# SENP6 Maintains Mitochondrial Homeostasis by Regulating Mitochondrial Protein Import Through deSUMOylation of TOM40

**DOI:** 10.1002/advs.202503408

**Published:** 2025-07-29

**Authors:** Liubing Hu, Jianshuang Li, Haolin Guo, Lei Su, Peina Dong, Juan Huang, Yanyan Liu, Xinjie Liu, Zhenhuan Luo, Wei Xiong, Zhenyu Ju, Qinghua Zhou, Hao Wang, Wenjun Wang

**Affiliations:** ^1^ The First Affiliated Hospital Key Laboratory of Regenerative Medicine of the Ministry of Education Jinan University Guangzhou Guangdong 510632 China; ^2^ The College of Life Science and Technology Jinan University Guangzhou Guangdong 510632 China; ^3^ Department of Orthopaedics Guangzhou Red Cross Hospital Faculty of Medical Science Jinan University Guangzhou Guangdong 510220 China; ^4^ The Sixth Affiliated Hospital of Jinan University (Dongguan Eastern Central Hospital) Jinan University Dongguan Guangdong 523067 China; ^5^ The Biomedical Translational Research Institute Health Science Center (School of Medicine) Jinan University Guangzhou Guangdong 510632 China; ^6^ MOE Key Laboratory for Membraneless Organelles and Cellular Dynamics Hefei National Science Center for Physical Sciences at Microscale and University of Science and Technology of China School of Life Sciences/Division of Biomedical Sciences Hefei Anhui 230026 China; ^7^ The Institute of Aging and Regenerative Medicine Jinan University Guangzhou Guangdong 510632 China

**Keywords:** Mitochondrial protein import, SENP6, SUMOylation, TOM40, TOM complex

## Abstract

SUMOylation, a reversible post‐translational modification, regulates various mitochondrial processes, including biogenesis, dynamics, mitophagy, and the mitochondrial unfolded protein response. Although SUMOylation is shown to be triggered by mitochondrial protein import failure in yeast, its impact on mammalian mitochondrial protein import remains unclear. Here, it is demonstrated that SENP6 knockdown‐induced SUMOylation causes loss of mitochondrial proteostasis, which impairs mitochondrial morphology and function. Mechanistically, SENP6 knockdown dampens TOM complex assembly by SUMOylating TOM40, thereby hindering the mitochondrial protein import process, including TOM40 precursor, and ultimately disrupts mitochondrial homeostasis. Additionally, it is observed that CCCP treatment resulted in a decrease of SENP6 within mitochondria fraction, accompanied by increased TOM40 SUMOylation in the brains of 3×Tg‐Alzheimer's disease (AD) mice or Aβ_1‐42_ peptide‐stimulated cells. Collectively, the results suggest that Aβ_1‐42_ accumulation may enhance TOM40 SUMOylation by suppressing SENP6, thereby impairing mitochondrial homeostasis through protein import failure and potentially contributing to the pathological process of AD. This study elucidates the role of TOM40 SUMOylation/deSUMOylation in regulating the mitochondrial import process during mitochondrial stress.

## Graphical abstract

SUMOylation regulates mitochondrial processes, but its impact on protein import remains unclear. TOM40 is identified, a mitochondrial outer membrane channel protein, as a substrate of deSUMOylase SENP6. TOM40 SUMOylation disrupts outer membrane complex assembly, inhibits protein import, and compromises mitochondrial homeostasis. Similar mechanisms are observed in AD models. This study reveals SUMOylated TOM40's role in mitochondrial stress‐responsive protein import regulation.

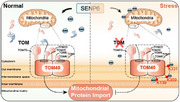

## 1 Introduction

Mitochondria are essential organelles with various functions, including ATP production, calcium regulation, and metabolic equilibrium. Approximately 99% of mitochondrial proteins are encoded by nuclear genes, synthesized as cytosolic precursors, and imported through specialized machinery.^[^ ^1^ ^]^ Beyond protein translocation, the mitochondrial protein import systems integrate with a functional network encompassing mitochondrial morphology, dynamics, bioenergetics, functions, and interactions with other organelles.^[^ ^2^ ^]^ Consequently, import defects disrupt mitochondrial homeostasis contributing to a range of diseases, including cancer, cardiovascular diseases, and neurodegenerative disorders.^[^ ^3^ ^]^ The translocase of the outer membrane (TOM) complex acts as the gateway for mitochondrial precursor proteins at the outer membrane. Translocation through TOM activates sorting mechanisms directing proteins to their target compartments.^[^ ^1^ ^]^ As the central pore component of the TOM complex, TOM40 interacts with other subunits (TOM5, TOM6, TOM7, TOM20, TOM22, and TOM70) to form the complex, facilitating the import of most mitochondrial proteins into the mitochondria.^[^ ^4^, ^5^ ^]^ Genome‐wide association studies (GWAS) have revealed an association between Single Nucleotide Polymorphisms (SNPs) within *TOMM40* and mitochondrial dysfunction, as well as metabolic syndrome.^[^ ^6^, ^7^ ^]^ Dysfunction of TOM40 has also been implicated in neurodegenerative disorders such as Alzheimer's disease, Parkinson's disease, and Huntington's disease.^[^ ^8^, ^9^ ^]^ Multiple studies have found that *TOMM40* SNPs, in conjunction with APOE ε3 and APOE ε4, are linked to late‐onset Alzheimer's disease (LOAD) or related endophenotypes.^[^ ^10^ ^]^ Furthermore, evidence suggests that TOM40 is associated with AD hippocampal atrophy, cognitive performance, and susceptibility risks,^[^ ^11^ ^]^ independently of APOE. While LOAD brains show elevated TOM40 mRNA,^[^ ^12^ ^]^ peripheral blood studies report reduced levels persisting at 1–2 year follow‐ups,^[^ ^13^, ^14^ ^]^ suggesting mitochondrial import dysregulation contributes to AD pathology.

SUMOylation dynamically modifies proteins via covalent SUMO conjugation to lysine residues, mediated by E1‐activating, E2‐conjugating, and E3‐ligase enzymes, with deSUMOylation regulated by SENPs.^[^ ^15^ ^]^ Unlike ubiquitination‐mediated degradation, SUMOylation modulates protein activity, localization, and stability, influencing gene expression, genome integrity, cell cycle, apoptosis, and immunity.^[^ ^16^ ^]^ Recent research has revealed that SUMOylation/ deSUMOylation are involved in regulating mitochondrial biogenesis,^[^ ^17^ ^]^ dynamics,^[^ ^18^ ^]^ mitophagy,^[^ ^19^ ^]^ and mitochondrial unfolded protein response (UPRmt).^[^ ^20^ ^]^ In addition, mitochondrial protein import failure and impaired proteostasis trigger SUMOylation of mitochondrial proteins in yeast,^[^ ^21^ ^]^ suggesting that SUMOylation may also play a role in the mitochondrial protein import system. SENP6 specifically cleaves polySUMO2/3 chains,^[^ ^22^ ^]^ regulating DNA repair,^[^ ^23^ ^]^ apoptosis,^[^ ^24^ ^]^ senescence,^[^ ^25^ ^]^ centromere assembly,^[^ ^26^ ^]^ inflammation,^[^ ^27^ ^]^ microglial polarization,^[^ ^28^ ^]^ angiogenesis,^[^ ^29^ ^]^ and nuclear architecture.^[^ ^30^ ^]^ Its dysfunction associates with lymphoma,^[^ ^31^ ^]^ laminopathies,^[^ ^32^ ^]^ and skeletal aging.^[^ ^25^ ^]^ However, SENP6's roles in mitochondrial homeostasis remain unexplored.

In the present study, we aim to investigate the role and underlying molecular mechanisms of SENP6 in the regulation of mitochondrial function and homeostasis. We observed that SENP6 knockdown (KD) adversely impacts mitochondrial morphology, proteostasis, and function. Mechanistically, SENP6 KD inhibits the assembly of the TOM complex by SUMOylating TOM40, consequently disrupting the mitochondrial protein import process, including TOM40 precursor. Additionally, we noted that CCCP treatment induces a decrease of SENP6 in the mitochondrial fraction, which resulting in deSUMOylation of TOM40 and mitochondria dysfunction. Furthermore, we observed decreased SENP6 and increased TOM40 SUMOylation in the brains of AD mice and Aβ_1‐42_ stimulated SH‐SY5Y cells. Our results suggest that Aβ accumulation may enhance the SUMOylation of TOM40 by downregulating SENP6, which leads to impaired mitochondrial protein import and triggers mitochondria dysfunction, thereby potentially enhancing the pathological process of AD.

## 2 Results

### 2.1 SENP6 Knockdown Impairs Mitochondrial Morphology and Function

**Figure 1:**
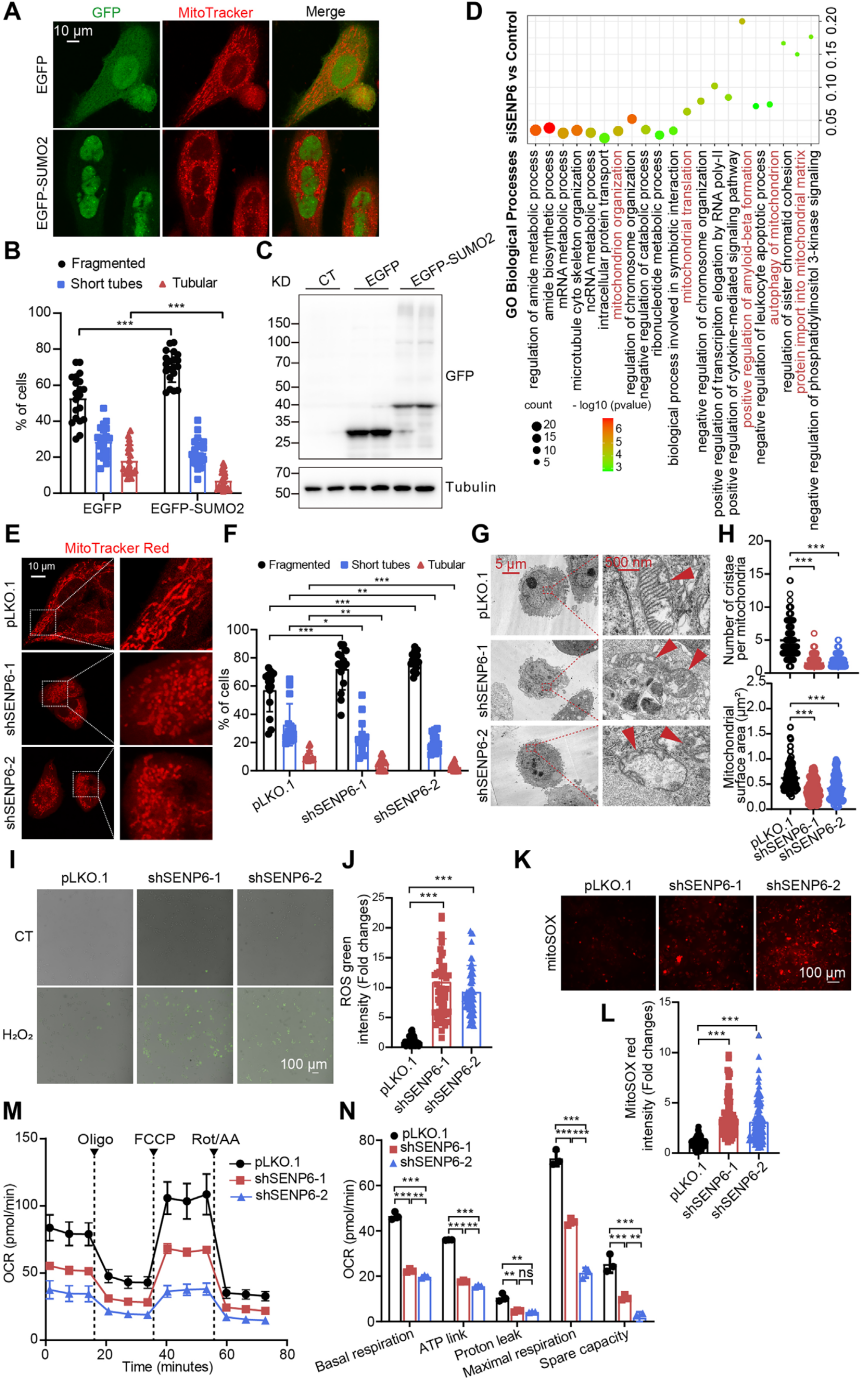
SENP6 knockdown impairs mitochondrial morphology and function. A,B) HeLa cells were transiently transfected with EGFP‐SUMO2 or EGFP (green), and mitochondria were labeled by MitoTracker (red). The percentages of tubular, short‐tube‐shaped, or fragmented mitochondria were determined based on MitoTracker staining (n = 20 cells, ^***^ *P* < 0.001). scale bar 10 µm). C) EGFP or EGFP‐SUMO2 expression levels were detected by western blot. D) Gene Ontology (GO) Biological Processes analysis was performed on the whole‐cell proteome data of control or siSENP6‐treated HeLa cells. E,F) Mitochondria in HeLa cells (pLKO.1, shSENP6‐1, or shSENP6‐2) were labeled with MitoTracker (red). The percentages of tubular, short‐tube‐shaped, or fragmented mitochondria were determined based on MitoTracker staining (n = 15 cells; ^*^ *P <* 0.05; ^**^ *P* < 0.01; ^***^ *P* < 0.001). The scale bars, 10 µm. G,H) The TEM was used to examine mitochondria in HeLa cells (pLKO.1, shSENP6‐1 or shSENP6‐2). The red arrows indicate mitochondria. The scale bars, 500 nm. The mitochondrial surface area (n = 110 mitochondria from pLKO.1, n = 142 mitochondria from shSENP6‐1, n = 114 mitochondria from shSENP6‐2 ^***^ *P* < 0.001), and the cristae number per mitochondrion (^***^ *P* < 0.001) were analyzed. I,J) The intracellular status of DCFH‐DA was observed using a fluorescence microscope in HeLa cells (pLKO.1, shSENP6‐1, or shSENP6‐2). The cells were exposed to H_2_O or H_2_O_2_ (500 µm) for 1 h. K,L) The mitochondrial MitoSOX red status was visualized using a fluorescence microscope in HeLa cells (pLKO.1, shSENP6‐1, or shSENP6‐2). M,N) The oxygen consumption rate (O_2_ consumption rate, OCR) was measured in HeLa cells (pLKO.1 or shSENP6) by a Seahorse cell energy metabolism analyzer. Oligomycin (Oligo), 1 µm; carbonyl cyanide‐4‐(trifluoromethoxy) phenylhydrazone (FCCP), 1 µm; Rotenone (Rot)/Antimycin A (AA), 0.5 µm. Parameters such as basal respiration, ATP link, proton leak, maximal respiration, and spare capacity were analyzed (n = 3 biologically independent samples; ^*^ *P* < 0.05; ^**^ *P* < 0.01; ^***^ *P* < 0.001).

Our previous study demonstrated that ginkgolic acid, a SUMOylation inhibitor, impairs mitochondrial function by decreasing mitochondrial biogenesis and promoting FUNDC1‐dependent mitophagy.^[^ ^33^ ^]^ We further observed EGFP‐SUMO2 overexpression promoted mitochondria fragmentation (**Figure** **1**A–C), while SUMO1 overexpression has no morphological impact (Figure S1, Supporting Information), suggesting SUMO2/3‐specific SUMOylation critically regulates mitochondrial homeostasis.

SENP6 has been reported to function as a rheostat of chromatin residency in genome maintenance and chromosome dynamics through a proteomic profiling study.^[^ ^23^ ^]^ Upon reanalysis of their whole‐cell proteome data, we found significant downregulation of numerous Gene Ontology (GO) Biological Processes related to mitochondria in the SENP6‐depleted cells (Figure 1D), including mitochondrial translation (Log_10_(P) = −4.09638), mitochondrion organization (Log_10_(P) = −4.70801), protein import into mitochondrial matrix (Log_10_(P) = −3.1008), and autophagy of mitochondrion (Log_10_(P) = −2.8036). Thus, we focus on investigating the role of SENP6‐regulated SUMOylation in mitochondrial homeostasis. SENP6 knockdown (KD) induced mitochondrial fragmentation (Figure 1E,F), cristae loss, and mitochondrial area reduction (Figure 1G,H). Furthermore, SENP6 KD notably reduced the levels of several mitochondrial proteins, including mitochondrial out membrane proteins (TOM40, TOM20, and TOM70), mitochondrial inner membrane proteins (TIM23), and mitochondrial matrix proteins (Hsp60, p32, and SDHA) in HeLa and SiHa cells (Figure S2A–D, Supporting Information). Rescue experiments revealed that overexpression of wild‐type (FLAG‐SENP6‐WT) but not catalytically inactive SENP6 (FLAG‐SENP6‐c1030a) restored mitochondrial proteins such as TOM20, TIM23, and p32 (Figure S2E,F, Supporting Information). Collectively, these results indicate the pivotal role of SENP6 in maintaining mitochondrial morphology and proteostasis.

Next, we evaluated the mitochondrial function in SENP6 KD cells. A substantial accumulation of cellular ROS levels was also observed in SENP6 KD cells (Figure 1I,J). Moreover, the level of mitochondrial ROS in SENP6 KD cells, measured by MitoSOX indicator, was notably increased compared to pLKO.1 cells (Figure 1K,L). To assess whether SENP6 KD impairs mitochondrial function, we measured oxygen consumption rates (OCRs) to detect the oxidative phosphorylation respiratory capacity. We found that compared to pLKO.1 cells, the basal respiration, ATP link, proton leak, maximal respiration, and spare capacity were significantly reduced in SENP6 KD cells (Figure 1M,N). Collectively, these results demonstrate that SENP6 KD promotes the impairment of mitochondrial morphology and function.

### 2.2 SENP6 Knockdown Enhances the SUMOylation of TOM40

To investigate SENP6's regulatory mechanism in mitochondrial function, we focused on its enzymatic activity given its crucial role in mitochondrial protein homeostasis (Figure S2E,F, Supporting Information). Analysis of published SENP6 targets identified TOM40 as a SUMO2/3 substrate in SENP6‐depleted cells (Figure S3, Supporting Information),^[^ ^23^ ^]^ TOM40 forms the central channel of the TOM complex, essential for mitochondrial protein import. Consistent with this, SENP6 depletion reduced TOM40 levels in HeLa and SiHa cells (Figure S2A–D, Supporting Information). This data triggered us to investigate whether SENP6 regulates mitochondrial function via TOM40. SENP6 is predominantly localized to the nucleus; however, recent studies have indeed confirmed its presence in the cytoplasm of diverse cell types.^[^ ^34^, ^35^ ^]^ In this study, we employed co‐staining of SENP6 with mitochondrial markers‐TOM20 for the outer membrane and TIM23 for the inner membrane‐in HeLa cells. Our findings revealed that a minor subset of SENP6 is localized to mitochondria under normal conditions (Figure S4A, Supporting Information). Furthermore, through subcellular fractionation, we identified that this mitochondrial localization of SENP6 was partial and was reduced in response to CCCP‐induced mitochondrial stress (Figure S4B, Supporting Information). We also noted that SENP6 interacts with TOM40, and this interaction was diminished after CCCP treatment, as observed in Figure S4C–E (Supporting Information). Collectively, these data suggest that under normal conditions, a fraction of SENP6 is localized to mitochondria, where it interacts with TOM40. However, mitochondrial localization and interaction are suppressed under mitochondrial stress.

**Figure 2:**
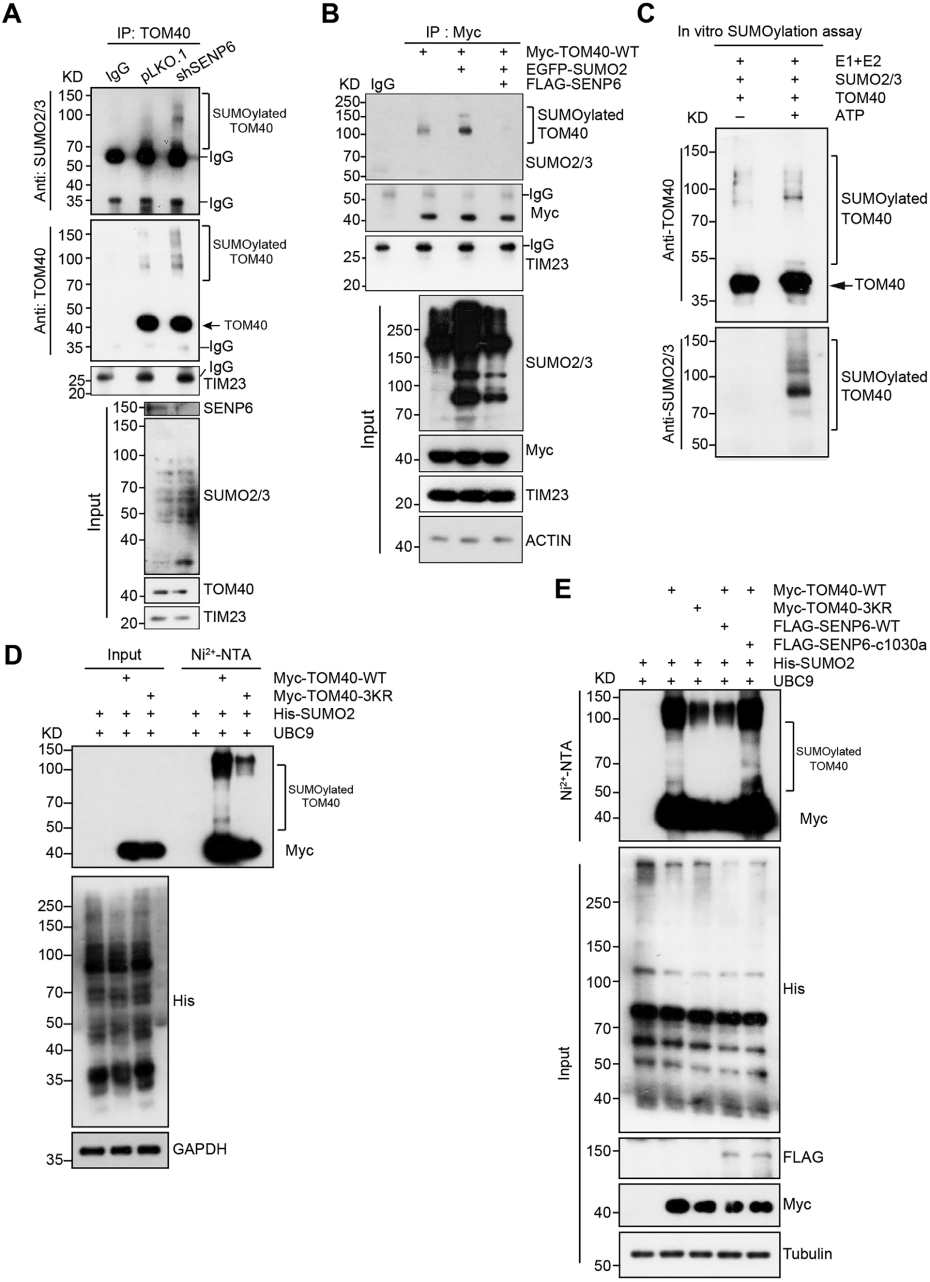
TOM40 is deSUMOylated by SENP6. A) Immunoprecipitation assays against endogenous TOM40 in HeLa cells (pLKO.1 or shSENP6) were carried out to detect SUMOylation with antibodies against SUMO2/3 or TOM40. TIM23 as a negative control. B) 293T cells were transiently transfected with Myc‐TOM40‐WT alone, Myc‐TOM40‐WT and EGFP‐SUMO2, or together with FLAG‐SENP6. The SUMOylation of TOM40 was detected by immunoprecipitation assays. TIM23 as a negative control. C) The recombinant TOM40 was incubated with E1, E2, SUMO2, SUMO3, and ATP in vitro at 37 °C for 1 h, and the reaction was terminated with SDS loading buffer. The samples prepared above were analyzed by Western blotting with SUMO2/3 and TOM40 antibodies as indicated. D) 293T cells were co‐transfected with Myc‐TOM40‐WT or Myc‐TOM40‐3KR, together with His‐SUMO2 and UBC9. SUMO2 conjugates were pulled down by Ni‐NTA affinity isolation. E) 293T cells were co‐transfected with Myc‐TOM40‐WT or Myc‐TOM40‐3KR, His‐SUMO2, and FLAG‐tagged WT SENP6 or the catalytic‐deficient mutant of SENP6 (SENP6‐c1030a). A Ni^2+^‐NTA affinity pull‐down assay was used for SUMOylation detection.

Given SENP6's role as a SUMO protease, we hypothesized that the interaction between SENP6 and TOM40 may regulate the SUMOylation of TOM40. Endogenous IP experiments were first performed and showed elevated TOM40 SUMOylation in SENP6 KD versus control cells (**Figure** **2**A). Additionally, compared to TOM40 overexpressed alone, co‐overexpression of TOM40 with EGFP‐SUMO2 shown increased SUMOylated TOM40. Most critically, the SUMOylation of TOM40 was abolished in the presence of overexpressed SENP6 (Figure 2B). The SUMOylation of TOM40 was further confirmed by the in vitro SUMOylation assay (Figure 2C). The in situ PLA assay also shown a direct in situ interaction of SUMO2/3 with TOM40, which was increased by SENP6 KD (Figure S5A,B, Supporting Information). Collectively, these data demonstrated SENP6 deSUMOylated TOM40.

To identify TOM40 SUMOylation sites, we used four prediction tools, including SUMOplot Analysis Programme (Abgent), JASSA,^[^ ^36^ ^]^ GPS‐SUMO,^[^ ^37^ ^]^ and Ron Hay's SUMO motif search tool, revealing three evolutionarily conserved lysine residues (K139, K309, and K331) across from mouse, rat, bovine to human (Figure S5C, Supporting Information). To validate these findings, we constructed the TOM40 mutants in which the lysine residues K139, K309, and K331 were either individually replaced with arginine (K139R, K309R, K331R) or all three simultaneously (3KR). Through Ni‐NTA affinity isolation assay, we found that the wide‐type TOM40 could be SUMOylated in the presence of His‐SUMO and UBC9, while SUMOylation of TOM40 was significantly reduced when overexpressed the TOM40‐3KR (Figure 2D,E). In contrast, mutating each lysine residue singly only resulted in a modest decrease in TOM40 SUMOylation (Figure S5D, Supporting Information). Furthermore, wide‐type SENP6, but not its catalytically dead mutant (c1030a), efficiently deSUMOylated TOM40 (Figure 2E). Collectively, these findings indicate that TOM40 can be SUMOylated at K139, 309 and 311, and that SENP6 plays a role in facilitating the deSUMOylation of TOM40.

### 2.3 SUMOylation of TOM40 Phenocopies SENP6 Knockdown‐Mediated Mitochondrial Dysfunction

**Figure 3:**
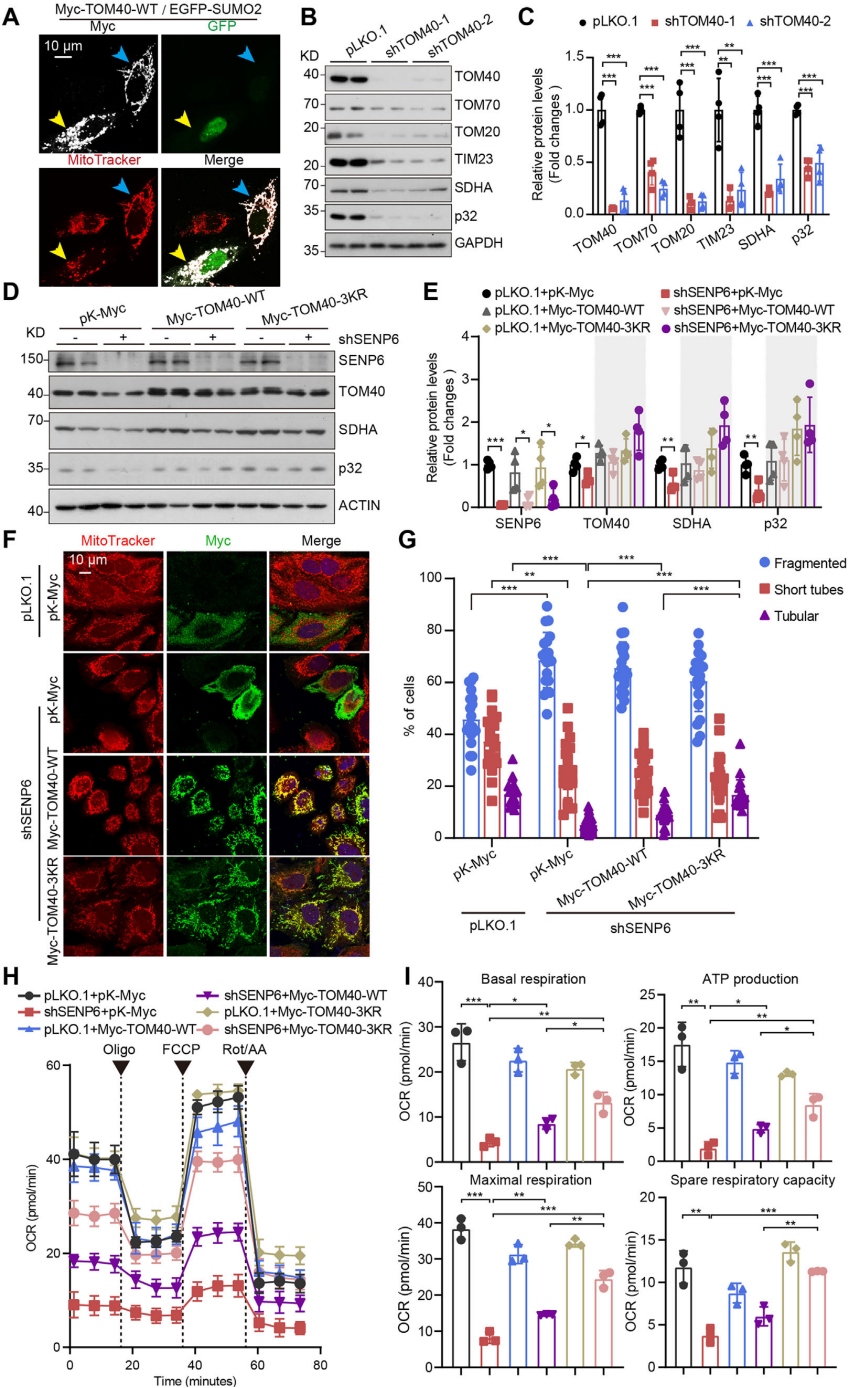
SUMOylation of TOM40 phenocopies SENP6 knockdown‐mediated mitochondrial dysfunction. A) Myc‐TOM40‐WT and EGFP‐SUMO2 were transiently co‐overexpressed, and the mitochondrial morphological changes were observed through immunofluorescence in HeLa cells. Myc, white; MitoTracker, red; GFP, green. Myc‐TOM40‐WT alone, blue arrow; co‐transfected with Myc‐TOM40‐WT and EGFP‐SUMO2, yellow arrow. Scale bar, 10 µm. B,C) The expression levels of mitochondria‐related proteins were analyzed by western blot in HeLa cells (pLKO.1, shTOM40‐1 or shTOM40‐2), (n = 4 biologically independent samples, ^**^ *P* < 0.01, ^***^ *P* < 0.001). D,E) HeLa cells (pLKO.1 or shTOM40) transiently overexpressed pK‐Myc, Myc‐TOM40‐WT or Myc‐TOM40‐3KR, and the expression levels of SENP6, mitochondria‐related proteins TOM40, SDHA, and p32 were analyzed by western blot (n = 4 biologically independent samples; ^*^ *P* < 0.05; ^**^ *P* < 0.01; ^***^ *P* < 0.001). F,G) pK‐Myc, Myc‐TOM40‐WT, or Myc‐TOM40‐3KR were overexpressed in shSENP6 HeLa cells, and mitochondrial morphological changes were observed using immunofluorescence. Myc, green; MitoTracker, red. Scale bar, 10 µm. The percentages of tubular, short‐tube‐shaped, or fragmented mitochondria were determined based on TOM20 staining (n = 20 cells; ^**^ *P* < 0.01; ^***^ *P* < 0.001; Myc‐TOM40‐3KR versus pK‐Myc for tubular). H,I) Seahorse cell energy metabolism analyzer was used to assess the OCR in HeLa cells (pLKO.1 or shSENP6). pK‐Myc, Myc‐TOM40‐WT, or Myc‐TOM40‐3KR was transiently overexpressed in these HeLa cells. Oligo concentration, 1 µm; FCCP concentration, 1 µm; AA/ Ret concentration, 0.5 µm. Maximal respiration and spare capacity were quantified (n = 3 biologically independent samples; ^*^ *P* < 0.05; ^**^ *P* < 0.01; ^**^ *P* < 0.001).

Next, we sought to investigate the contribution of TOM40 SUMOylation toward mitochondrial morphology and function. The examination of mitochondrial morphology was initially conducted in HeLa cells co‐transfected with Myc‐TOM40‐WT and EGFP‐SUMO2. Overexpression of Myc‐TOM40‐WT alone did not cause obvious mitochondrial fragmentation (indicated by the blue arrow), while cells co‐transfected with Myc‐TOM40‐WT and EGFP‐SUMO2 showed mitochondria fragmentation (indicated by the yellow arrow) (**Figure** **3**A), indicating that SUMOylated TOM40 may be the key point for impaired mitochondria morphology, which is also found in SENP6 KD cells.

We next asked whether TOM40 could influence mitochondrial proteostasis. The knockdown of TOM40 considerably decreased levels of several mitochondrial proteins (Figure 3B,C), which was also observed in SENP6 KD cells. Importantly, the overexpression of both Myc‐TOM40‐WT and Myc‐TOM40‐3KR partially reverted SENP6 KD‐mediated reduction of mitochondrial proteins (Figure 3D,E), indicating the critical role of TOM40 on SENP6‐regulated mitochondrial proteostasis. Myc‐TOM40‐WT or Myc‐TOM40‐3KR overexpression significantly increased the relative ratio of tubular mitochondria and partially rescued mitochondrial fragmentation in SENP6 KD cells (Figure 3F,G). Moreover, compared to the wide‐type TOM40, the non‐SUMOylated TOM40, Myc‐TOM40‐3KR increases more tubular mitochondria in SENP6 KD cells (Figure 3F,G).

Next, we investigate whether SENP6‐regulated deSUMOylation of TOM40 influences mitochondrial respiration capacity. The measurement of oxygen consumption rates (OCRs) revealed that the overexpression of TOM40 induced a partial increase in basal respiration, ATP production, and maximal respiration in SENP6 KD cells (Figure 3H,I). In contrast, overexpression of the non‐SUMOylated TOM40‐3KR variant significantly enhanced basal respiration, ATP production, maximal respiration, and spare respiratory capacity in SENP6 KD cells. Overexpression of TOM40‐3KR significantly enhanced the four respiratory capacity indicators of mitochondria compared to overexpression of TOM40‐WT (Figure 3H,I). Notably, the maximal respiration and spare respiratory capacity returned to levels similar to those of control cells following the overexpression of TOM40‐3KR (Figure 3H,I). These findings suggest that SENP6 KD causes mitochondrial dysfunction via TOM40, with the SUMOylation of TOM40 playing a pivotal role in this dysregulation.

### 2.4 SENP6 Knockdown‐Mediated TOM40 SUMOylation Inhibits the Assembly of TOM Complex

**Figure 4:**
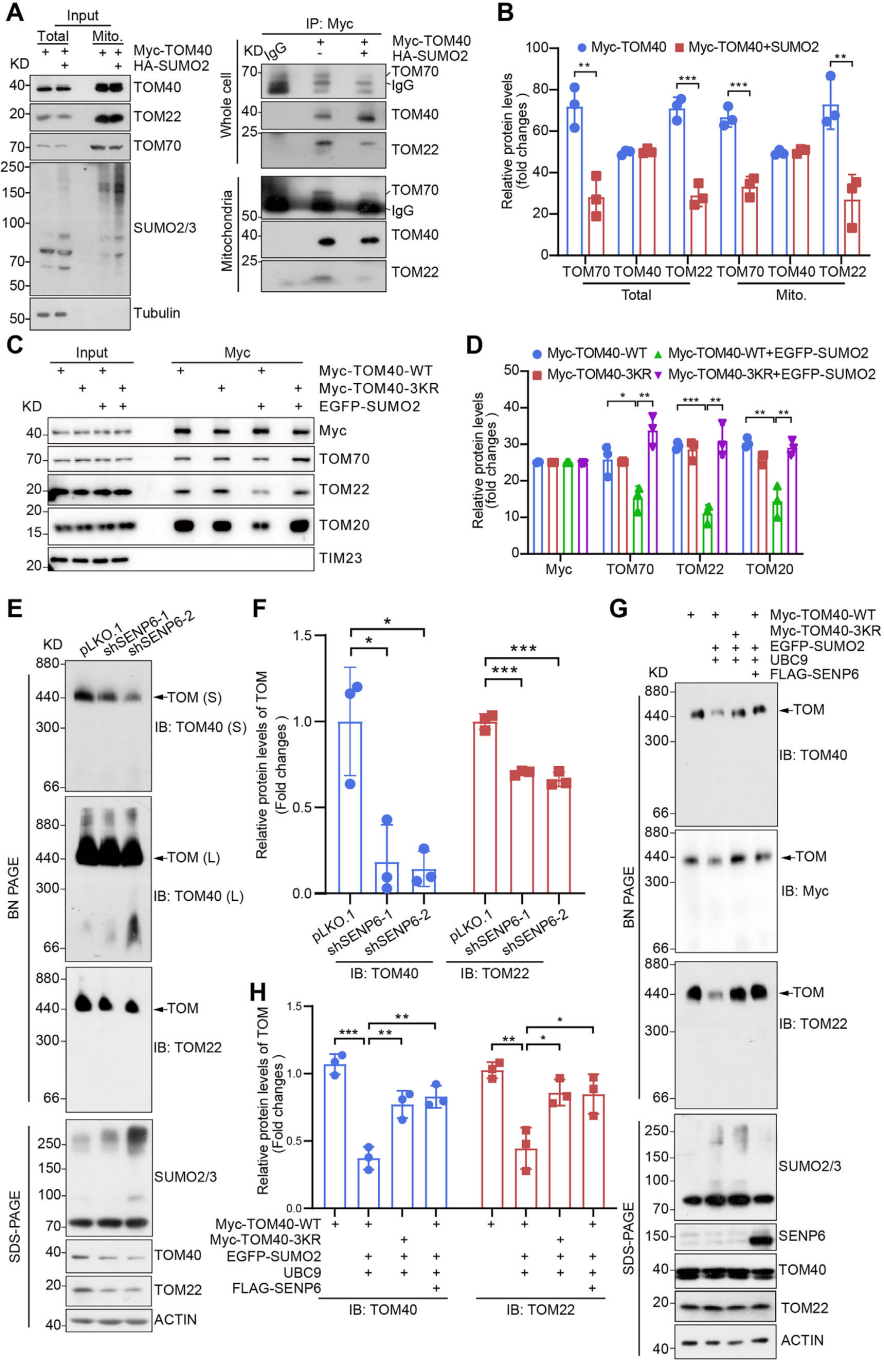
SENP6 knockdown‐mediated TOM40 SUMOylation inhibits the assembly of the TOM complex. A,B) Whole or isolated mitochondrial proteins from 293T cells transiently transfected with Myc‐TOM40‐WT and HA‐SUMO2, or Myc‐TOM40‐WT alone, were immunoprecipitated with anti‐Myc to examine interactions between exogenous TOM40 and subunits of the TOM complex (TOM70 and TOM22). (n = 3 biologically independent samples; ^**^ *P* < 0.01; ^***^ *P* < 0.001) C,D) 293T cells transiently transfected with Myc‐TOM40‐WT or Myc‐TOM40‐3KR together with EGFP‐SUMO2, or Myc‐TOM40‐WT, Myc‐TOM40‐3KR alone, were immunoprecipitated with anti‐Myc beads to examine interactions between exogenous Myc‐TOM40 and subunits of TOM complex (TOM70, TOM22, or TOM20). TIM23 as a negative control. (n = 3 biologically independent samples; ^*^ *P* < 0.05; ^**^ *P* < 0.01; ^***^ *P* < 0.001). E,F) Outer mitochondrial membrane proteins from HeLa cells were isolated using digitonin and loaded onto a blue native‐PAGE to assess the assembly of the TOM complex, which was detected using anti‐TOM40 or anti‐TOM22 antibodies (top), (n = 3 biological replicates; ^*^ *P* < 0.05). The expression of SUMO2/3 was confirmed by western blot (bottom). G,H) Outer mitochondrial membrane proteins from 293T cells transfected with Myc‐TOM40‐WT alone, or EGFP‐SUMO2 and UBC9 together with Myc‐TOM40‐WT, or Myc‐TOM40‐3KR, or Myc‐TOM40‐WT and FLAG‐SENP6 were isolated using digitonin. The assembly of TOM complex was assessed by subjecting the proteins to blue native‐PAGE and detected using anti‐TOM40, anti‐Myc, or anti‐TOM22 antibodies (top), (n = 3 biological replicates; ^*^ *P* < 0.05; ^**^ *P* < 0.01). The overexpression of SENP6, SUMO2/3, and TOM40 was confirmed by western blot (bottom).

SUMOylation has been reported to affect the stability, subcellular location, and protein–protein‐interaction of target proteins.^[^ ^38^ ^]^ By interaction with other TOM subunits, TOM40 assembles the TOM complex to import most of nuclear‐encoded proteins into mitochondria.^[^ ^39^ ^]^ Thus, we wondered whether the SUMOylation of TOM40 influenced the interactions between TOM40 with other TOMs, and affected TOM complex assembly. The overexpression of Myc‐TOM40‐WT with or without HA‐SUMO2 did not alter the expression levels of TOM22 and TOM70 but reduced the interactions between TOM40 with TOM22 and TOM70 in whole lysis and mitochondrial fraction of 293T cells (**Figure** **4**A,B). Conformably, co‐expression of Myc‐TOM40‐WT and EGFP‐SUMO2 inhibited the interactions between TOM40 and TOM22, TOM70, or TOM20, whereas Myc‐TOM40‐3KR significantly restored these interactions (Figure 4C,D). TOM40 interacts with small TOM subunits, including TOM5, TOM6, and TOM7 to constitute the early TOM complex.^[^ ^4^ ^]^ Our investigation revealed that the SUMOylation of TOM40 does not perturb its interaction with these small TOM subunits (Figure S6, Supporting Information). To confirm that SENP6 KD‐mediated SUMOylation of TOM40 repressed TOM complex assembly, non‐denaturing electrophoresis was utilized to resolve high‐relative‐molecular‐weight mitochondrial protein complexes. Our results revealed that the proportion of TOM40 and TOM22 incorporated into the TOM complex was prominently reduced in SENP6 KD cells (Figure 4E,F). Furthermore, we sought to determine whether the un‐SUMOylated form of TOM40 (Myc‐TOM40‐3KR) or deSUMOylation of TOM40 by SENP6 could restore the TOM complex assembly. Indeed, the upregulation of SUMOylation induced by the transfection of EGFP‐SUMO2 and UBC9 decreased the proportion of TOM40 and TOM22 incorporated into the TOM complex, while the ectopic expression of FLAG‐SENP6 and Myc‐TOM40‐3KR significantly rescued the incorporation of TOM40 and TOM22 into the TOM complex (Figure 4G,H). Collectively, these data show that SUMOylation of TOM40 attenuates the interaction between TOM40 with TOM subunits (TOM20/22/70), dampening TOM complex assembly, which is negatively regulated by SENP6.

### 2.5 SENP6 Maintains Mitochondrial Proteostasis by Regulating Mitochondrial Protein Import

As the major entry channel for mitochondrial proteins, TOM complex is also a key regulator for mitochondrial protein quality control.^[^ ^1^ ^]^ To investigate whether SENP6 maintains proteostasis via import regulation, we used a combination of established methods. These included the visualization of mitochondrial‐targeted green fluorescent protein (mitoGFP^[^ ^40^ ^]^ and Su9‐EGFP^[^ ^41^ ^]^), the analysis of mitochondrially targeted Su9‐DHFR‐FLAG fusion protein,^[^ ^42^ ^]^ as well as the application of the split‐GFP system.^[^ ^42^ ^]^

**Figure 5:**
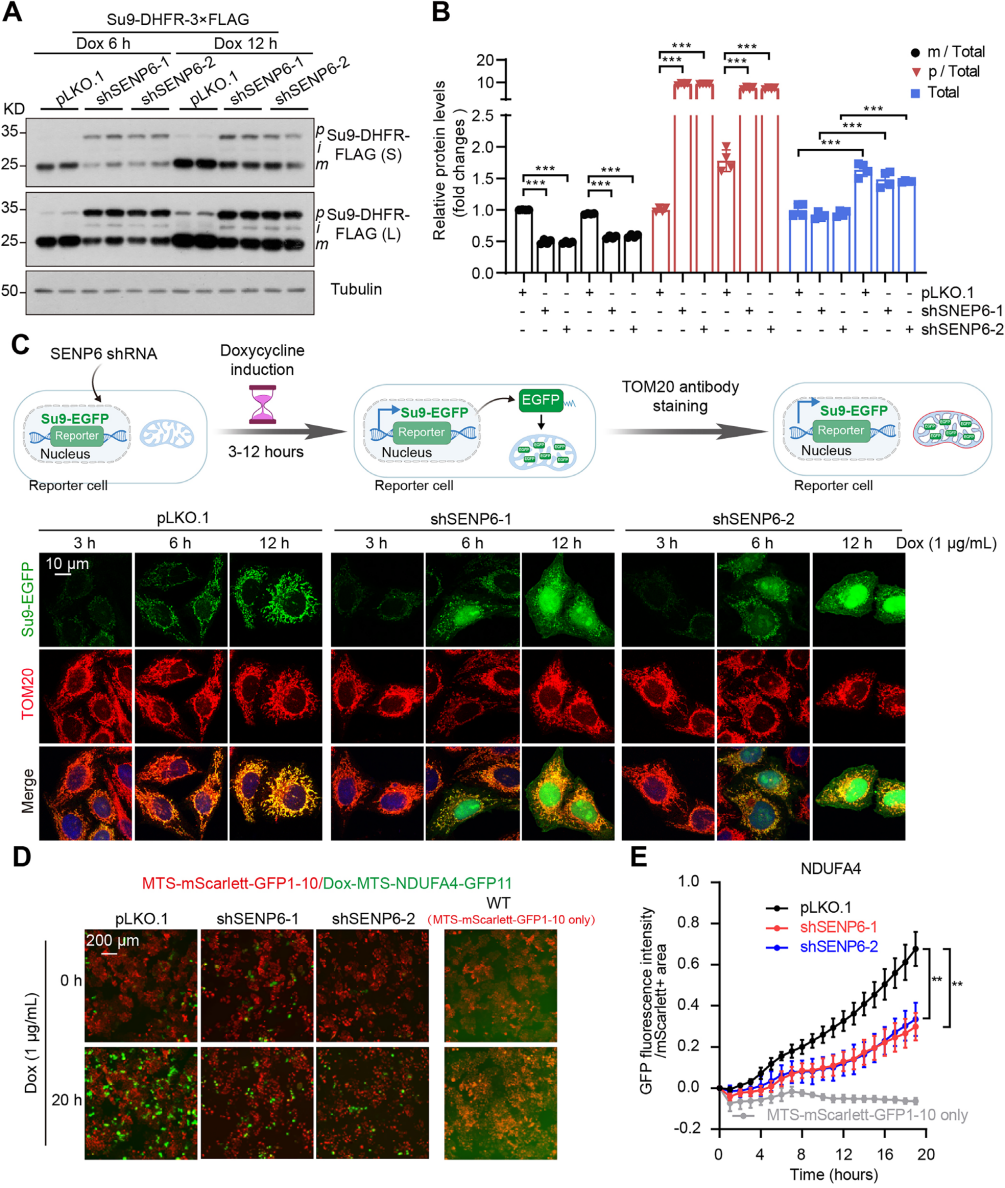
Mitochondrial protein import is reduced in SENP6 KD cells. A,B) A stable HeLa cell line expressing Dox‐Su9‐DHFR‐FLAG was established through lentiviral infection and puromycin selection. Expression of Su9‐DHFR‐FLAG was detected in WT and SENP6 KD cells after being induced with Dox for 6 or 12 h. Quantification of total Su9‐DHFR‐FLAG (*p* + *i* + *m*), and mature (m), intermediate (i), and precursors (*p*) Su9‐DHFR‐FLAG normalized to total. n = 4 biologically independent samples; ^***^ *P* < 0.001). C) A stable HeLa cell line expressing Dox‐Su9‐EGFP was established through lentiviral infection and puromycin selection. Expression of Su9‐EGFP was induced with Dox for 3, 6, or 12 h. TOM20 was used to label the mitochondria (red), and the colocalization of green and red fluorescence was observed. D) A stable HeLa cell line expressing MTS‐Scarlett‐GFP1‐10 was established through lentiviral infection and puromycin selection. Time‐lapse imaging of MTS‐Scarlett‐GFP1‐10 (Red) and Dox‐NDUFA4‐GFP11 in (Green) pLKO.1 or SENP6 KD cells (Scale bar, 200 µm). E) Quantification of GFP fluorescence intensity generated over time, normalized by mitochondrial area (mScarlett signal) for each import substrate tested. (n = 3–6 biologically independent samples; 4 fields per sample were collected every h for 20 h; ^**^ *P* < 0.01).

First, we utilized a fusion protein of mitoGFP (the mitochondrial‐targeting sequence of Aconitase 2 together with GFP),^[^ ^40^ ^]^ in which the mitochondrial targeting sequence is cleaved within the mitochondria to release the mature fluorescent protein. The intensity of mitoGFP appeared to decrease within the mitochondria of SENP6 KD cells (Figure S7A, Supporting Information). To biochemically probe the mitochondrial protein import process in more detail, we utilized Su9‐DHFR‐FLAG containing Neurospora crassa F0‐ATPase subunit 9 signal sequence and 3×FLAG tag, enabling immunoblot detection of precursor [p], intermediate [i], and mature [m] forms via matrix protease cleavage.^[^ ^42^ ^]^ Dox‐inducible Su9‐DHFR‐FLAG and Su9‐EGFP systems were utilized for a time‐resolved assay. Stable expression of Su9‐DHFR‐FLAG in HeLa cells revealed SENP6 KD reduced mature substrate levels by ≈40% versus pLKO.1 controls (**Figure** **5**A,B). Consistently, Su9‐EGFP predominantly localized to mitochondria in control cells across Dox timepoints (Figure 5C), SENP6 KD cells exhibited progressive cytosolic accumulation (Figure 5C; Figure S7B, Supporting Information). To dynamically monitor import kinetics, we employed a time‐resolved assay based on the split‐GFP system,^[^ ^42^, ^43^ ^],^ constitutively expressed MTS‐mScarlett‐GFP1‐10 (mitochondria‐localized) and Dox‐inducible NDUFA4‐GFP11. The MTS‐mScarlett‐GFP1‐10 was stably expressed in HeLa cells and was found to colocalize with mitochondria (Figure 5D). The mitochondrial import of NDUFA4 was monitored through Dox‐induced expression of NDUFA4‐GFP11 and time‐lapse imaging of GFP fluorescence over a 20‐h period. Cells co‐expressing both MTS‐mScarlett‐GFP1‐10 and the import substrate NDUFA4‐GFP11 showed GFP fluorescence that colocalized with mitochondria, while mitochondria of cells expressing only MTS‐mScarlett‐GFP1‐10 showed red fluorescence (Figure 5D). Quantitative analysis revealed significantly impaired NDUFA4 import efficiency in SENP6 KD cells (Figure 5E), confirming SENP6's essential role in mitochondrial protein import. In the Su9‐EGFP‐HeLa cell line, the defect in mitochondrial protein import was partially remedied by the expression of wild‐type TOM40. However, TOM40‐3KR exhibited a more effective rescue of mitochondrial protein import compared to wild‐type TOM40 (Figure S7C, Supporting Information).

We also observed upregulation of SUMO2/3 levels and downregulation of several mitochondrial proteins in Dox‐induced SENP6 KD cells, which is consistent with the stable SENP6 KD cells (Figure S8A, Supporting Information). The intensity of mitoGFP was significantly attenuated in a dose‐dependent manner upon Dox treatment (Figure S8B, Supporting Information). Additionally, stable or Dox‐induced SENP6 KD markedly inhibited the colocalization of Su9‐EGFP with mitochondria (Figures S7B and S8C, Supporting Information). Collectively, these data suggest that the deficiency of SENP6 repressed mitochondrial protein import via the SUMOylation of TOM40.

### 2.6 Pathological Relevance and Conservation of TOM40 SUMOylation

**Figure 6:**
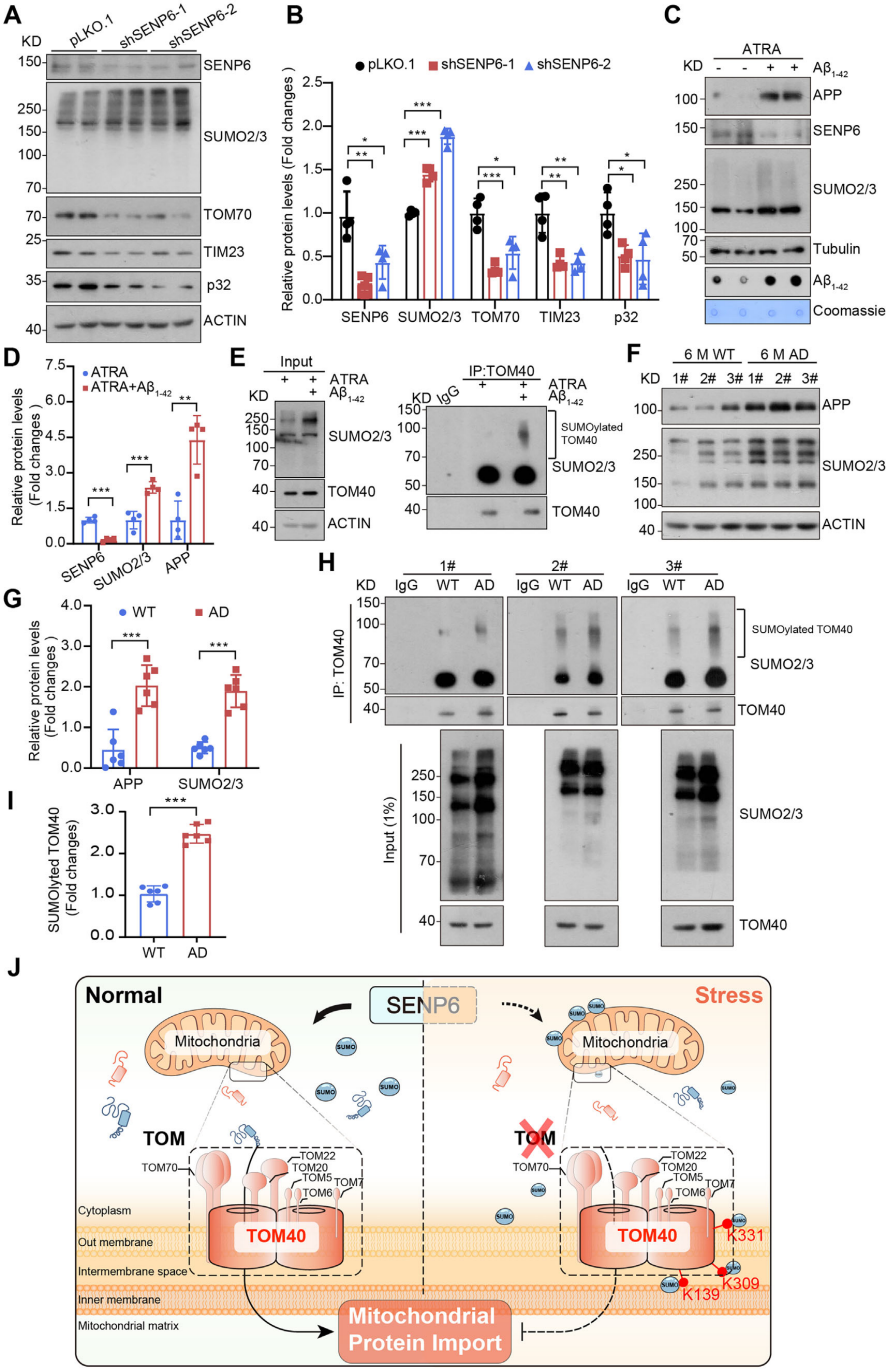
Physiological and pathological relevance and conservation of TOM40 SUMOylation. A,B) Western blot was conducted to assess the expression of SENP6, SUMO2/3, and mitochondrial‐related proteins TOM70, TIM23, and p32 in SH‐SY5Y cells (pLKO.1, shSENP6‐1, or shSENP6‐2), (n = 4 biologically independent samples; ^*^ *P* < 0.05; ^**^ *P* < 0.01; ^***^ *P* < 0.001). C,D) After treatment with ATRA (10 µm) for 14 days, the expression levels of APP, SENP6, and SUMO2/3 were analyzed by western blot, and the expression levels of Aβ_1‐42_ were analyzed by dot blot in SH‐SY5Y cells treated with Aβ_1‐42_ (5 µm, 48 h), (n = 4 biologically independent samples; ^**^ *P* < 0.01; ^***^ *P* < 0.001). E) After treatment with ATRA (10 µm) for 14 days, immunoprecipitation assays conducted against endogenous TOM40 in Aβ_1‐42_ (5 µm, 48 h) treated SH‐SY5Y cells, detecting SUMOylation with anti‐SUMO2/3 antibodies. F,G) Western blot was used to analyze the expression of APP/Aβ and SUMO2/3 in the cortex of WT or 3×Tg‐AD mice (n = 3 mice; ^***^ *P* < 0.001). H,I) Immunoprecipitation assays were performed against endogenous TOM40 in the cortex of WT or 3×Tg‐AD mice to detect SUMOylation with anti‐SUMO2/3 antibodies (n = 6 mice; ^***^ *P* < 0.001). J) Schematic diagram of SENP6 KD‐induced TOM40 SUMOylation impaired mitochondrial protein import.

Next, we wonder what's the pathological function of SENP6 and SENP6‐mediated deSUMOylation of TOM40. The GO‐BP analysis of proteomic data shows that SENP6 may regulate amyloid‐beta formation (Log_10_(P) = −4.713175012) (Figure 1D). It has been reported that *TOMM40* SNPs are associated with LOAD risk,^[^ ^11^, ^44^, ^45^ ^]^ cognitive performance,^[^ ^11^ ^]^ and hippocampal atrophy.^[^ ^46^ ^]^ And mitochondrial dysfunction is one of the risk factors for AD.^[^ ^47^ ^]^ All these drive our attention and force us to investigate whether SENP6 and TOM40 SUMOylation affect AD progress. First, we confirmed the downregulation of the mitochondrial critical proteins caused by SENP6 KD in the neuronal cell line SH‐SY5Y (**Figure** **6**A,B). SH‐SY5Y cells differentiated with ATRA (all‐trans‐retinoic acid) were utilized to simulate in vitro Aβ‐induced AD‐like damage in neuronal cells. We found that, after differentiation with ATRA, Aβ_1‐42_ peptide treatment decreased SENP6 expression and increased the levels of SUMO2/3 and SUMOylated TOM40 (Figure 6C–E; Figure S9A,B, Supporting Information).

To establish the relevance of this mechanism in AD mice, we evaluated the levels of TOM40 SUMOylation in the cortex of 6‐month‐old 3×Tg‐AD mice versus age‐matched WT mice. The significant accumulation of Aβ was affirmed in the hippocampus and cortex of mice (Figure S9C, Supporting Information). Consistent with the cell model, the upregulation of SUMO2/3 and SUMOylated TOM40 were also detected in the cortex of AD mice (Figure 6F–I). Taken together, the SUMOylation of TOM40 was increased in AD mice cortex and Aβ_1‐42_ treated SH‐SY5Y cells, indicating that the SUMOylation of TOM40 may associate with AD in the mice model.

In summary, our study uncovers that SENP6 KD enhances SUMOylation of TOM40, a pivotal mechanism that inhibits mitochondrial protein import, resulting in impaired proteostasis and mitochondria dysfunction. Mechanistically, SENP6 KD hinders the assembly of TOM complex by promoting SUMOylation of TOM40, which disrupts the import process of mitochondrial protein, including TOM40 precursor, and perturbs mitochondrial homeostasis (Figure 6J).

## 3 Discussion

SUMOylation and deSUMOylation play crucial roles in regulating various aspects of mitochondrial biology, including biogenesis,^[^ ^17^ ^]^ dynamics,^[^ ^18^ ^]^ mitophagy,^[^ ^19^ ^]^ and mitochondrial unfolded protein response (UPRmt).^[^ ^20^ ^]^ SENP6, a protease that removes poly‐SUMO2/3 modifications, is primarily localized in the nucleus, although it has also been observed in the cytoplasm of various cell types.^[^ ^34^, ^35^ ^]^ Our study identified a minor fraction of SENP6 present in mitochondria under normal conditions (Figure S4A, Supporting Information). We found that treatments with CCCP or Aβ_1‐42_ suppressed mitochondrial localization of SENP6, resulting in increased SUMOylation of TOM40 (Figure 6E–I). Through the knockdown of SENP6, we discovered that the absence of SENP6 enhanced SUMO2/3 modification of TOM40, leading to mitochondrial fragmentation, elevated reactive oxygen species (ROS), and compromised oxidative phosphorylation function (Figure 1; Figure S2, Supporting Information). Mechanistic analysis revealed that SENP6 facilitates deSUMOylation of specific lysine residues (K139, K309, and K331) on TOM40, which is necessary for maintaining interactions between TOM40 and other TOM subunits, such as TOM20, TOM22, and TOM70 (Figure 4C,D). The disruption of these interactions hampers TOM complex assembly, thereby impairing mitochondrial protein import and overall mitochondrial function. These findings underscore the critical role of SENP6‐mediated deSUMOylation of TOM40 in maintaining mitochondrial homeostasis, particularly under conditions of mitochondrial stress induced by CCCP or in the context of AD pathology.

TOM40, the central pore‐forming subunit of the mitochondrial TOM complex, mediated more than 90% mitochondrial protein import. Among seven TOM complex subunits (TOM40, 22, 20, 70, 5, 6, 7), TOM40 was uniquely essential for mitochondrial function and viability.^[^ ^48^, ^49^ ^]^ The lysine residues K309 and K331 of TOM40 are in close proximity to the TOM22 subunit,^[^ ^4^, ^5^ ^]^ and their SUMOylation may disrupt the interaction between TOM40 and TOM22, thereby impairing TOM complex assembly. Our data indicate that SUMOylation of TOM40 significantly inhibits the interaction between TOM40 and TOM22, as well as TOM20 and TOM70. In contrast, the non‐SUMOylated TOM40‐3KR variant (K139, 309, 331 mutated to R139, 309, 331) restores these interactions, even in the presence of EGFP‐SUMO2 (Figure 4C). We hypothesize that SUMOylation at K309 and K331 may sterically hinder or alter the conformation of TOM40, affecting its ability to interact with other subunits. The lysine residue K139, on the other hand, is relatively distant from other TOM subunits. The SUMOylation of TOM40 at K139 could lead to a conformational change that affects its interactions with other subunits.

The assembly of TOM complex is a dynamic process, consisting of three main stages: assembly I, where the precursor TOM40 is folded and inserted into the SAM complex; assembly II, involving the assembly of small TOM subunits (TOM5/6/7) onto TOM40 and the disassociation of SAM from TOM; and the formation of the TOM core complex, where TOM22 is recruited to the TOM core complex.^[^ ^4^ ^]^ The TOM core complex is ≈440 kDa, while the assembly II complex is ≈100 kDa, containing TOM40 and small TOM subunits but not TOM22, TOM20, or TOM70.^[^ ^4^, ^5^ ^]^ Our study demonstrates that SENP6 knockdown leads to SUMOylation of TOM40, which diminishes the interaction between TOM40 and TOM22/TOM20/TOM70 (Figure 4C). This suggests that SUMOylation of TOM40 may impair the late stages of TOM complex assembly, particularly the core complex assembly and the subsequent integration of TOM20 and TOM70 into the TOM complex. Critically, our data show that SUMOylation of TOM40 does not affect the interaction between TOM40 and the small TOM subunits, such as TOM5/6/7, which form the assembly II complex (≈100 kDa) (Figure S6, Supporting Information). This implies that SUMOylation of TOM40 does not disrupt the formation of the assembly II complex but rather hinders the final step of TOM core complex assembly by preventing the recruitment of TOM22/20/70. Considering the dynamic equilibrium between the 440 kDa functional trimer and the 100 kDa resting dimer, as described by Shiota et al.,^[^ ^5^ ^]^ we think that impairment in the late stages of assembly would result in an accumulation of the 100 kDa assembly II complex. This is consistent with our observations, where we detected an increase in the 100 kDa TOM complex in SENP6 KD cells (Figure 4E). Collectively, these data indicate that the loss of SENP6‐mediated SUMOylation of TOM40 at K139, K309, and K311 represses its interaction with TOM20 /22 / 70, thereby inhibiting the assembly of the TOM complex.

Like most nuclear‐encoded mitochondrial proteins, TOM40 is transcribed in the nucleus, translated into a precursor protein in the cytosol, imported into mitochondria, and ultimately assembled into the functional TOM complex.^[^ ^4^, ^50^ ^]^ Our data demonstrating the SUMOylation of TOM40 through various methodologies (Figure 2A–E; Figure S5A,B, Supporting Information). Mutagenesis studies have identified lysine residues K139, K309, and K331 as specific sites for SUMOylation (Figure 2D,E; Figure S5D, Supporting Information). Nonetheless, we recognize a technical limitation in our study: our existing methodologies do not provide direct visualization of SUMOylated TOM40 within the fully assembled TOM complex. Overcoming this limitation would likely necessitate the dissociation of intact complexes and ultra‐high‐resolution structural analysis, which currently presents insurmountable technical challenges. Regarding the stage‐specific SUMOylation of TOM40, it is likely that TOM40 SUMOylation may occur at multiple stages. Given that the lysine residues in the TOM40 precursor are exposed, it is plausible that they may be more susceptible to SUMOylation. Additional research is needed to determine the exact regulatory steps and conditions. Moreover, the inhibition of mitochondrial protein precursor import due to TOM40 SUMOylation could potentially lead to a reduced import of the precursor form of TOM40 itself. This may represent one of the molecular mechanisms contributing to the exacerbation of subsequent mitochondrial dysfunction.

Despite the presence of two SUMOylation sites (K139, K309) of TOM40 within the intermembrane space (IMS),^[^ ^51^ ^]^ existing literature indicates that SUMO2/3 can modify matrix‐localized proteins such as SDHA,^[^ ^52^ ^]^ indicating their extensive distribution within mitochondria. Although most identified SUMO E3 ligases are localized within the nucleus, a mitochondria‐localized SUMO E3 ligase, known as Mitochondrial‐anchored protein ligase (MAPL/MUL1), has been recently discovered. MUL1 is recognized for its role in regulating mitochondrial morphology and localization.^[^ ^53^, ^54^ ^]^ Furthermore, MUL1 has been identified as a pro‐SUMOylation E3 ligase for NLRP3, which localizes to mitochondria, with SENP6 functioning as a negative regulator.^[^ ^55^ ^]^ Our co‐IP assays revealed a robust interaction between TOM40 and MUL1 (Figure S10A, Supporting Information). Furthermore, Ni^2^⁺‐NTA pulldown assays demonstrated that MUL1 significantly enhanced TOM40 SUMOylation (Figure S10B, Supporting Information). These results indicate that the mitochondria‐localized E3 ligase MUL1 promotes TOM40 SUMOylation through direct interaction. SENP6 co‐localizes with the outer membrane protein TOM20, inner membrane protein TIM23, and interacts directly with TOM40 (Figure S4, Supporting Information). These findings provide compelling evidence that the spatial proximity enables SUMO2/3 molecules, along with the regulatory enzymes (MUL1, SENP6), to access the relevant lysine residues on TOM40, thereby establishing the structural and spatial foundation for its SUMOylation regulation.

TOM complex‐mediated mitochondrial protein import maintains both mitochondrial and cellular fitness.^[^ ^2^, ^41^, ^56^ ^]^ Nuclear‐encoded mitochondrial proteins typically contain classical mitochondrial targeting signals, such as prosequences, which are cleaved post‐import to facilitate proper mitochondrial function.^[^ ^56^ ^]^ In our study, we observed that the short‐term induction of Su9 in SENP6 KD cells led to a suppression of both the intermediate and mature forms of Su9, while the precursor form was increased (Figure 5A,B). Furthermore, SENP6 KD enhanced the cytosolic localization of Su9 following Dox induction, depending on the duration of induction (Figure 5C). These findings indicate that SENP6 KD does not impact the expression of the mitochondrial precursor protein but rather inhibits its import into the mitochondria. Following import failure, mitochondrial precursor proteins remain locate in the cytosol, where they undergo degradation via the proteasome pathway.^[^ ^57^, ^58^ ^]^ Thus, in our study, we prefer to demonstrate that SENP6 KD not only promoted the instability of TOM40 but also repressed TOM40's protein–protein‐interaction with other TOMs, which resulted in impaired TOM complex assembly and mitochondrial protein import. In turn, the deficiency of mitochondrial protein import results in mitochondrial proteins reduction, mitochondrial fragmentation, and mitochondrial dysfunction. SUMOylation/deSUMOylation contributed to Alzheimer's pathogenesis through modification of core AD‐associated proteins, such as Aβ, APP, tau, BACE1, GSK3β, and JNK.^[^ ^59^, ^60^ ^]^ Elevated SUMO‐1 levels occurred in AD patient cortex^[^ ^61^ ^]^ and plasma^[^ ^62^ ^]^ and Tg2576 mouse brains.^[^ ^63^ ^]^ SUMO isoforms differentially modulate pathology: SUMO‐3 inhibits amyloid aggregation while SUMO1 promotes APP aggregation.^[^ ^64^, ^65^ ^]^ BACE1 SUMO‐1 modification specifically enhanced Aβ production and contributed to AD neuropathology.^[^ ^66^ ^]^ Our data revealed SENP6 (SUMO2/3‐specific deconjugate) dysfunction exhibits increased SUMO2/3 in AD models (Aβ_1‐42_‐treated SH‐SY5Y cells and brain tissues from 6‐month‐old 3×Tg‐AD mice) (Figure 6C–G). However, decreased SUMO‐2/3 levels were detected in older APP mice at 17 months of age, whereas increased levels of SUMO1, Ubc9, and SENP1 levels were observed in younger APP mice at 3 and 6 months of age.^[^ ^63^ ^]^ These data indicates that SUMOylation contributes to the development of AD and SUMO‐1/SUMO‐2/3 have diverse functions in the pathogenesis of AD in a course‐dependent manner.

Aβ peptides accumulate both on and inside mitochondria in AD, impairing protein import through TOM complex blockade and directly inducing mitochondrial dysfunction linked to cognitive decline, though Aβ_1‐42_ and TOM complex binding alone suffice to inhibit import.^[^ ^67^, ^68^ ^]^ Our data reveal that exposure to the Aβ_1‐42_ peptide suppresses the overall expression of SENP6, consequently augmenting TOM40 SUMOylation (Figure 6C–E). Notably, heightened SUMOylation of TOM40 was also detected in the brains of AD mice. This alteration may underlie the impairment of mitochondrial protein import and subsequent mitochondrial dysfunction, thus unveiling a novel mechanism by which Aβ exacerbates the pathophysiological processes of AD through SENP6‐dependent regulation of mitochondrial homeostasis. However, whether SENP6 directly regulates the pathogenesis of AD via TOM complex‐mediated mitochondrial protein import needs further study.

## 4 Experimental Section

### Cell Culture

293T (SCSP‐502), HeLa (SCSP‐504), SiHa (SCSP‐5058) and SH‐SY5Y (SCSP‐5014) cells were obtained from the Shanghai Cell Bank, Type Culture Collection Committee, Chinese Academy of Sciences and cultured in Dulbecco's modified Eagle's medium (DMEM, 4.5 g L^−1^ glucose, 4 mm L‐glutamine, Gibico, C11995500BT) containing 10% fetal bovine serum (FBS, sigma, F0193), 100 units mL^−1^ penicillin and 100 mg mL^−1^ streptomycin (Gibico,15140‐122).

### Plasmid Construction

The gene encoding TOM40 was cloned into BamHI/EcoRI sites of pK‐Myc expression vectors using standard PCR‐based cloning strategies and verified by DNA sequencing. TOM40‐K139R, ‐K309R, ‐K331R, and ‐3KR plasmids were constructed by the Site‐Directed Mutagenesis kit (following the manufacturer's instructions). The gene encoding DHFR‐FLAG was cloned into Su9‐EGFP expression vectors using standard PCR‐based cloning strategies and verified by DNA sequencing (Angte Biotechnology, Guanzhou, China). The SUMO2 gene was inserted into the EGFP vector at the C‐terminal of the EGFP.

shRNA sequences from The RNAi Consortium collection (MISSION shRNA, Sigma, www.sigmaaldrich.com), which utilizes constitutive pLKO vector, can be ordered as oligos and cloned into pLKO‐Tet‐On.

For shRNA design, use BLOCK‐iTRNAi Designer (http://rnaidesigner.thermofisher.com/) to determine the two top‐scoring targets for SENP6. Two target sequences are listed in Table S1 (Supporting Information). The pLKO.1 vector was digested with EcoRI and AgeI, and then ligated with shRNA oligos by the T4 ligase.

The “all‐in‐one” system was used for the inducible expression of SENP6 shRNA. This construct was generated by sequential PCR‐based modification of the pLKO lentiviral vector. pLKO‐Tet‐On contains all the necessary cis‐elements for packaging, reverse transcription, and integration, which were required for subsequent production of the lentiviral particles. More importantly, this vector also contains all the necessary components for the inducible expression of shRNA in target cells. In the absence of tetracycline/doxycycline, shRNA expression was repressed by constitutively‐expressed TetR protein. Upon the addition of tetracycline/doxycycline to the growth media, shRNA expression was triggered, resulting in target gene knockdown.

### Lentivirus Package and Transfection

To generate lentiviral particles, it conducted co‐transfection of shSENP6 plasmids into 293T cells along with psPAX2 and pMD2.G, employing the polyethyleneimine (PEI) transfection reagent. The lentiviral supernatants were collected at 48‐ and 72‐h post‐transfection and subsequently used to infect HeLa, SiHa, and SH‐SY5Y cells. Following infection, the cells were subjected to selection in a medium containing 2 µg mL^−1^ of puromycin for three days.

### Mice

All animals were housed at room temperature (22°C ± 2°C), with a 12‐h light‐dark cycle with ad libitum access to food and water. Animals were housed 2–5 per cage. Animal care and experiments were performed in strict accordance with the ‘“Guide for the Care and Use of Laboratory Animals,” which was approved by the Experimental Animal Ethical Committee at Jinan University (Approval number: IACUC‐20210318‐06). Male WT and 3×Tg‐AD mice (DM11003) were purchased from Aniphe Biolaboratory (Jiangsu, China). The 3×Tg‐AD mice used in this study have a C57BL/6 genetic background and harbor three human mutant genes associated with familial Alzheimer's disease, namely APPSwe, tauP301L, and Psen1tm1 Mpm.^[^ ^69^ ^]^ Intracellular Aβ deposition becomes noticeable by 3–4 months, followed by the presence of extracellular Aβ deposition, impaired synaptic transmission, and long‐term potentiation (LTP) at 6 months, and hippocampal deposits of hyperphosphorylated tau were observed at 12–15 months. For this study, three 6‐month‐old male mice of the WT or 3×Tg‐AD were employed. All mice were starved for 12 h prior to sacrifice. Mice were euthanized under deep anesthesia (≈ 4% isoflurane), and the brain tissues were collected.

### Subcellular Fractionation and Mitochondria Isolation

Cells were collected and subjected to centrifugation at 400 × g for 5 min at 4 °C. The resulting cell pellet was washed once with PBS, followed by permeabilization using 250 µg mL^−1^ digitonin (Sigma, D141) in PBS for 10 min at 25 °C. Afterward, the cells were centrifuged at 10 000 × g for 10 min at 4 °C, and the resulting supernatants were collected into a fresh tube, representing the cytosolic fraction. Meanwhile, the pellets were reconstituted in a mitochondrial lysate buffer (containing 10 mm Tris‐HCl, pH 7.4, 150 mm NaCl, 2 mm EDTA, 0.2% Triton, 0.3% NP40, along with protease inhibitor cocktails, PMSF, and NEM) for 30 min on ice, and subsequently centrifuged at 10 000 × g for 20 min at 4 °C. This procedure yielded the supernatants as the mitochondrial fraction and the pellets as the nuclear fraction.

### Co‐Immunoprecipitation

Immunoprecipitation of endogenous SUMOylated TOM40 was performed with TOM40 (proteintech, 18409‐1‐AP). Immunoprecipitation of Myc‐TOM40 was performed with Myc‐tag (proteintech, 16286‐1‐AP). Protein–protein interactions were detected using anti‐Myc MagBeads (Yeasen, 20567ES03) or anti‐Flag NanoMagBeads (Fitgene, FI8201). Cells were collected in N‐Ethylmaleimide (NEM)‐PBS and then lysed with 4% SDS IP buffer (50 mm Tris‐HCl (pH 7.5), 150 mm NaCl, 1% Triton X‐100, 1 mM EDTA, 5% Glycerol and 4% SDS, protease inhibitor, and 20 mm NEM) with sonicated and boiling for 10 min. Subsequent dilution of SDS to 0.1% by 50 mm Tris‐HCl (pH 7.5). Protein concentrations were measured with a Pierce BCA protein assay kit (Thermo Scientific, 23225). Lysis proteins (2 mg) were used for the pull‐down procedure. After overnight immunocoprecipitation, 60 µL of 1× SDS was added to elute the bound proteins. Subsequently, the precipitates were boiled, and 20 µL of the eluted sample was loaded onto SDS‐PAGE gels for western blot analysis with primary (TOM40, SUMO2/3, Myc.ect) and secondary antibodies (Mouse Anti‐Rabbit IgG (Light‐Chain Specific) (D4W3E), 93702) or VeriBlot for IP Detection Reagent (ab131366).

### Western Blot

In western blot analyses, samples were lysed with RIPA buffer (Beyotime, P0013B) with protease inhibitors (PMSF, Protease Inhibitor Cocktail, and NEM) and sonicated. Then the protein concentrations were measured with a Pierce BCA protein assay kit. Total cell lysates were loaded onto SDS‐PAGE gels and transferred onto Hybond P–polyvinylidene difluoride membranes (PVDF, Millipore). The membrane was then blocked in tris‐buffered saline‐Tween (TBST) [50 mm Tris‐HCl (pH 8.0), 15 mm NaCl, 0.1% Tween 20] supplemented with 5% milk, followed by incubating with primary antibodies for 2 h at 25 °C or overnight at 4 °C. The primary and secondary antibodies used are shown in Table S2 (Supporting Information). Protein bands were evaluated using Quantity One 1‐D analysis software (Bio‐Rad, Hercules, CA). Protein levels were quantified relative to ACTB in the same sample, and the relative protein expression was normalized to the respective control group, which was set to 1.

### Immunofluorescence Staining

After treatment, for mitochondria labeling, cells were continued incubated in DEME media containing 50 nM MitoTracker Red (Invitrogen, M7513) for 30 min at the incubator. Then cells were fixed for 20 min using 4% paraformaldehyde, followed by a 10‐min permeabilization step with 0.1% Triton X‐100 in PBS. After three PBS washes, cells were blocked with a mixture of 2% BSA (Amresco, E588) and 10% FBS (Sigma, F0193) for 1 h at 25 °C. Subsequently, cells were incubated overnight at 4 °C with the following primary antibodies: anti‐TOM20 (Proteintech, 11802‐1‐AP, 1:400), Hsp60 (Proteintech, 15282‐1‐AP, 1:400), and Myc‐tag (Proteintech, 60003‐2‐Ig, 1:400). Following three PBST washes, cells were treated with appropriate secondary antibodies (Alexa Fluor 594‐AffiniPure goat anti‐rabbit (Jackson, 115‐585‐146) or Alexa Fluor 488‐AffiniPure goat anti‐mouse IgG (Jackson, 115‐545‐146)) for 1 h at room temperature, followed by DAPI counterstaining. Finally, images were taken and analyzed with a Leica TCS SP8 confocal microscope.

### Blue‐Native PAGE

Blue native polyacrylamide gel electrophoresis (BN‐PAGE) was performed to detect TOM complex. Cells were collected and permeabilized with 250 µg mL^−1^ digitonin (Sigma, D141) and the precipitates were suspended in cold 1×Native PAGE Sample Buffer containing 5 mg mL^−1^ digitonin and protease inhibitor cocktail/PMSF on ice for 15 min to solubilize the organelle proteins. After centrifugation (20 000×g, 30 min at 4 °C), the supernatants were collected, and determined the lysate protein concentration using BCA protein assay. 5% G‐250 sample additive was added, then the samples were loaded on a 4–15% RealPAGE native BN/CN gradient gel (Real‐Times Biotechnology, Beijing, China). After electrophoresis, proteins were transferred to the PVDF blotting membrane. After transfer, incubated the membrane in 8% acetic acid for 15 min to fix the proteins, washed with deionized water and air‐dry the membrane, and rewet the membrane with methanol, then probed proteins with specific antibodies against TOM40 (Proteintech, 18409‐1‐AP, 1:5000), TOM22 (Proteintech, 11278‐1‐AP, 1:2000) or Myc (Proteintech, 16286‐1‐AP, 1:5000) incubation followed by immunoblot analysis.

### Electron Microscopy

HeLa cells were cultured in 10‐cm dishes and subsequently harvested to be fixed with 2.5% glutaraldehyde for 12 h at 4 °C. Following fixation, cell monolayers underwent three PBS washes and were then post‐fixed using 1% osmium tetroxide in 0.1 m PBS for 2 h at room temperature. After a triple PBS wash, the samples were dehydrated through a series of ethanol solutions and embedded in EMBed 812 epoxy resin (SPI, 90529‐77‐4). Ultrathin sections (60 nm) were mounted on copper grids and stained with 2% uranyl acetate in saturated alcohol for 8 min, followed by exposure to 2.6% lead citrate for 8 min. Images were captured using a Hitachi HT7800 transmission electron microscope. The electron microscopy assays were conducted in collaboration with Servicebio (Wuhan, China).

### ROS Measurement

After treated with 500 µm H_2_O_2_ for 1 h, HeLa cells were harvested, washed with phosphate‐buffered saline (PBS, pH 7.2), and incubated with 10 µmol L^−1^ fluorescent probe DCFH‐DA (S0033S, Beyotime, China) for 0.5 h at 37 °C. And then ROS generation was measured by the fluorescence microscope (Nikon, Japan).

For mitochondrial ROS levels were measured using MitoSox (M36008, Thermo Fisher Scientific). In brief, cells were stained with 5 µm MitoSOX at 37 °C for 30 min and then analyzed by the fluorescence microscope (Nikon, Japan).

### Oxygen Consumption Rate (OCR)

Cells were plated in a XF96‐well microplate (Seahorse Bioscience) followed by OCR measurement at 37 °C using an XF96 Analyzer (Seahorse Bioscience) in accordance with the manufacturer's instructions. Then, 1 µm oligomycin, 1 µm FCCP, and 1 µm rotenone/antimycin from Seahorse XF Cell Mito Stress Test Kit (Agilent, 103015‐100) were delivered to detect the spare respiration capacity, maximal respiration capacity, and ATP productivity, respectively.

### Mitochondrial Import Imaging Assay

The mitochondrial import imaging assay was performed as described previously.^[^ ^45^ ^]^ A stable HeLa cell line was established expressing MTS‐mScarlett‐GFP1‐10 through lentiviral infection and puromycin selection for subsequent analysis. Then the stable cells were transfected with import substrate‐GFP11 for 24 h. Dox (2 mg mL^−1^) was added to the culture medium, and images were acquired using ImageXpress Micro Confocal live cell imaging system (Molecular Devices, San Jose, CA, USA). Four fields were collected every hour for 20 h, and images were analyzed on the MetaXpress version 6.6.1.42 (Molecular Devices) by normalizing the integrated intensity of GFP signal to the area of mScarlett signal. For each experiment, the data were normalized to the signal in wild‐type cells at 20‐h time point.

### In Vitro SUMOylation Assay

In vitro SUMOylation of TOM40 was carried out with a SUMOylation kit (BML‐UW8955‐0001, Enzo Life Sciences). The commercial purified TOM40 protein (Proteintech, Cat: Ag13065) was suspended in SUMOylation buffer (20 µL) containing E1, E2, SUMO2, SUMO3, and adenosine triphosphate (ATP). Following 1 h incubation at 37 °C, the reaction was terminated with SDS loading buffer. The samples prepared above were analyzed by Western blotting with SUMO2/3 and TOM40 antibodies as indicated.

### Dot‐Immunoblotting

After incubation with ATRT (10 µm) for 14 days, SH‐SY5Y cells were treated with Aβ_1‐42_ (2.5 or 5 µm for 48 h). Cells were collected and lysed with RIPA buffer (Beyotime, P0013B) with protease inhibitors (PMSF, Protease Inhibitor Cocktail) and sonicated. After centrifuging at 12 000×g, the supernatant protein was collected, and the concentrations were measured with a Pierce BCA protein assay kit. Total cell lysates were loaded onto PVDF membranes (Millipore). As described in the preceding text, immunoprecipitation of Myc‐TOM40 was performed with anti‐Myc MagBeads (Yeasen Biotechnology, 20567ES03). The eluted sample was loaded onto PVDF membranes. The membrane was then blocked in TBST supplemented with 5% milk, followed by incubating with Aβ_1‐42_ antibody (abcam, ab201060, 1:6000 diluted), TOM5 (Proteintech, 25607‐1‐AP, 1:1000), TOM6 (Proteintech, 16689‐1‐AP, 1:1000), or TOM7 (Proteintech, 15071‐1‐AP, 1:1000) overnight at 4 °C. After three successive 10‐min washes with TBST, the membranes were incubated with HRP‐Rabbit secondary antibody (Jackson Immunoresearch, Cat: 111‐035‐003, 1:8000 diluted) at room temperature for 2 h.

### Immunohistochemical Staining

Mouse brain tissue samples were fixed in 10% neutral buffered formalin, embedded in paraffin, and sectioned to 4 µm slides. After dewaxing and rehydration, tissue antigens were retrieved by heating the slides using citrate buffer or EDTA solution. Sections were incubated overnight with a rabbit Aβ_1‐42_ antibody (abcam, ab201060, 1:500 diluted) with primary antibodies at 4 °C overnight and with a biotinylated anti‐rabbit antibody (Vector Laboratories, BA‐1000). Positive staining was visualized by DAB substrate reaction (Vector Laboratories, SK‐4100) following the ABC kit protocol (Vector laboratories, PK‐6100), and co‐straining with hematoxylin.

### Ni^2+^‐NTA Affinity Isolation Assay

Ni2^+2^‐NTA pull‐down was used to purify the SUMOylation of TOM40 in the case of denaturation.^[^ ^52^ ^]^ 293T cells were transfected for 48 h with the indicated plasmids (Table S2, Supporting Information key resources table). Cells were collected in *N*‐Ethylmaleimide (NEM)‐PBS, and 25% of cells were used for RIPA buffer lysis and Western blot. Then the remainder was lysed with His‐lysis buffer (6 M guanidinium‐HCl, 0.1 m Na2HPO4/NaH2PO4, 0.01 m Tris/HCl, pH 8.0, 5 mm imidazole and 10 mm β‐mercaptoethanol, protease inhibitor and 20 mm NEM) with sonicated. Cell lysates (1 mg) incubated at 4 °C overnight with 50 µL Ni^2+^‐NTA agarose resin (Qiagen). The beads were successively washed for 5 min in each step at room temperature with 750 µL of each of the following buffers: washing buffer 1 (6 m Guanidine hydrochloride, 0.1 m Na_2_HPO_4_/NaH_2_PO_4_, 0.01 m Tris‐HCl, pH 8.0 and 10 mm b‐mercaptoethanol), washing buffer 2 (8 m urea, 0.1 m Na_2_HPO_4_/NaH_2_PO_4_, 0.01 m Tris‐HCl, pH 8.0 and 10 mm b‐mercaptoethanol), washing buffer 3 (8 m urea, 0.1 m Na_2_HPO_4_/NaH_2_PO_4_, 0.01 m Tris‐HCl, pH 6.3, 0.2% Triton X‐100 and 10 mm b‐mercaptoethanol) and washing buffer 4 (8 M urea, 0.1 m Na_2_HPO_4_/NaH_2_PO_4_, 0.01 m Tris‐HCl, pH 6.3, 0.1% Triton X‐100 and 10 mm b‐mercaptoethanol). After the last wash, His‐tagged sumoylateded products were eluted by incubating the beads in 100 µL of Elution buffer (200 mm imidazole, 0.15 m Tris‐HCl pH 6.7, 30% glycerol, 0.72 m b‐mercaptoethanol, 5% SDS) for 20 min at R.T. and assessed by western blot.

### In Situ Proximity Ligation Assay (PLA)

A proximity ligation assay was conducted utilizing the Duolink in situ Kit (Cambridge BioScience Ltd) in accordance with the manufacturer's protocol. In summary, following treatment, cells were fixed with 4% paraformaldehyde (PFA) for 20 min, permeabilized using 0.2% Triton X‐100 for 10 min, and subsequently blocked with Duolink blocking solution for 60 min at 37 °C. The cells were then incubated with anti‐TOM40 and anti‐SUMO2/3 or Flag antibodies, diluted in Duolink antibody diluent, for 2 h at room temperature. Following incubation, the cells were washed twice with wash buffer A and incubated with PLA probes (PLUS and MINUS) for 60 min at 37 °C. A ligation reaction was performed to facilitate the joining of hybrid connector oligos to the PLA probes for 30 min at 37 °C, followed by PLA signal amplification for 100 min at 37 °C. Finally, the cells underwent washing with buffer B and were stained with DAPI. Imaging was performed using a Leica TCS SP8 confocal microscope, and positive PLA puncta were analyzed using ImagePlus 6.0 software.

### Bioinformatic Analysis

Putative SUMOylation sites in TOM40 were identified using GPS‐SUMO, SUMOplot analysis (Abgent) software, JASSA, and Ron Hay's SUMO consensus motif search tool (www.lifesci.dundee.ac.uk/groups/ron_hay/pages/SumomotifQuery.html).

The Whole‐Cell Proteome in Control and SENP6‐depleted cells were download from the Supplemental Tables of Resource “The SUMO Isopeptidase SENP6 Functions as a Rheostat of Chromatin Residency in Genome Maintenance and Chromosome Dynamics” published in *Cell Reports* (2019, Volume 29, Issue 2, Pages 480‐494.e5); then GO analysis of the Differentially proteome significant changes was performed using Metascape website (https://metascape.org/gp/index.html#/main/step1).

### Statistics and Reproducibility

Unless specified otherwise, experiments were performed in biological triplicate, or sufficient sample sizes were involved. The results were reported as mean ± standard deviation (S.D.). All statistical analyses were performed using GraphPad Prism software. Differences among three or more means were evaluated using One‐way ANOVA, while differences between two means were assessed by using the two‐tailed, unpaired Student's t‐test in GraphPad Prism version 8.0. p values < 0.05 were considered statistically significant (^*^ *P* < 0.05, ^**^ *P* < 0.01, ^***^ *P* < 0.001, ^****^ *P* < 0.0001).

## Conflict of Interest

The authors declare no conflict of interest.

## Author Contributions

L.H. and J.L. contributed equally to this work; Q.Z., H.W., and W.W. jointly supervised this work. Q.H. Zhou, W.J. Wang, J.S. Li and H. Wang supervise this project. W. J. W., Q. H. Z., H. W., L. B. H. and J. S.L. designed experiments. L.B.H., J.S.L. and W.J.W. performed the experiments and data analysis. H. L. G., L. S., J. H., P. N. D., Y. Y. L., X. J. L., and Z. H. L. performed experiments. Z. Y. J., W. X., and H. W. critically read the paper and provided technical support. Q. H. Z., W. J. W., J. S. L., L. B. H. and, H. W. wrote the paper.

## Supporting information



Supporting Information

Supporting Information

## Data Availability

The data that support the findings of this study are available from the corresponding author upon reasonable request.
