# Supplementary material for: SENP6 Maintains Mitochondrial Homeostasis by Regulating Mitochondrial Protein Import Through deSUMOylation of TOM40
Source: Adv Sci (Weinh). 2025 Jul 29;12(40):e03408. doi: 10.1002/advs.202503408 (PMC12561365; doi:10.1002/advs.202503408)
Supplement: Supplementary file 1 — Supporting Information [file ADVS-12-e03408-s002.docx]

Supporting Information

**SENP6 Maintains Mitochondrial Homeostasis by Regulating Mitochondrial Protein Import Through deSUMOylation of TOM40**

*Liubing Hu, Jianshuang Li, Haolin Guo, Lei Su, Peina Dong, Juan Huang, Yanyan Liu, Xinjie Liu, Zhenhuan Luo, Wei Xiong, Zhenyu Ju, Qinghua Zhou^*^, Hao Wang^*^, Wenjun Wang^*^*

**Figure S1**

**
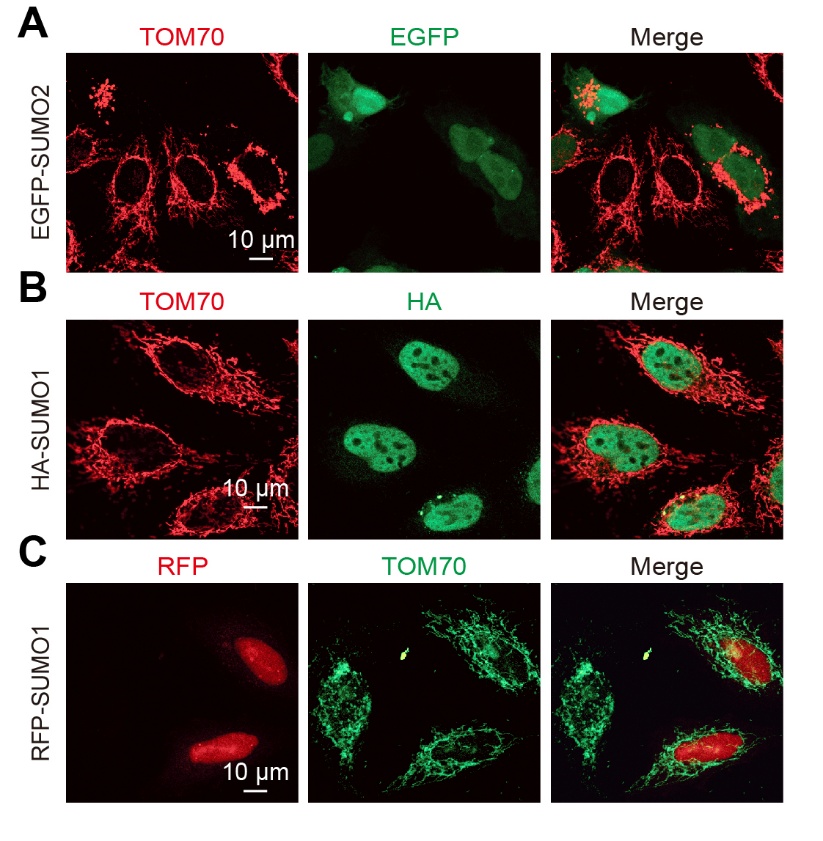
**

**Figure S1 The effect of SUMO1 or SUMO2 overexpression on mitochondrial morphology.** HeLa cells were transiently transfected with (A) EGFP-SUMO2 (green), (B) HA-SUMO1 (green) or (C) RFP-SUMO1 (red). Mitochondria were labeled using anti-TOM70.

**Figure S2**


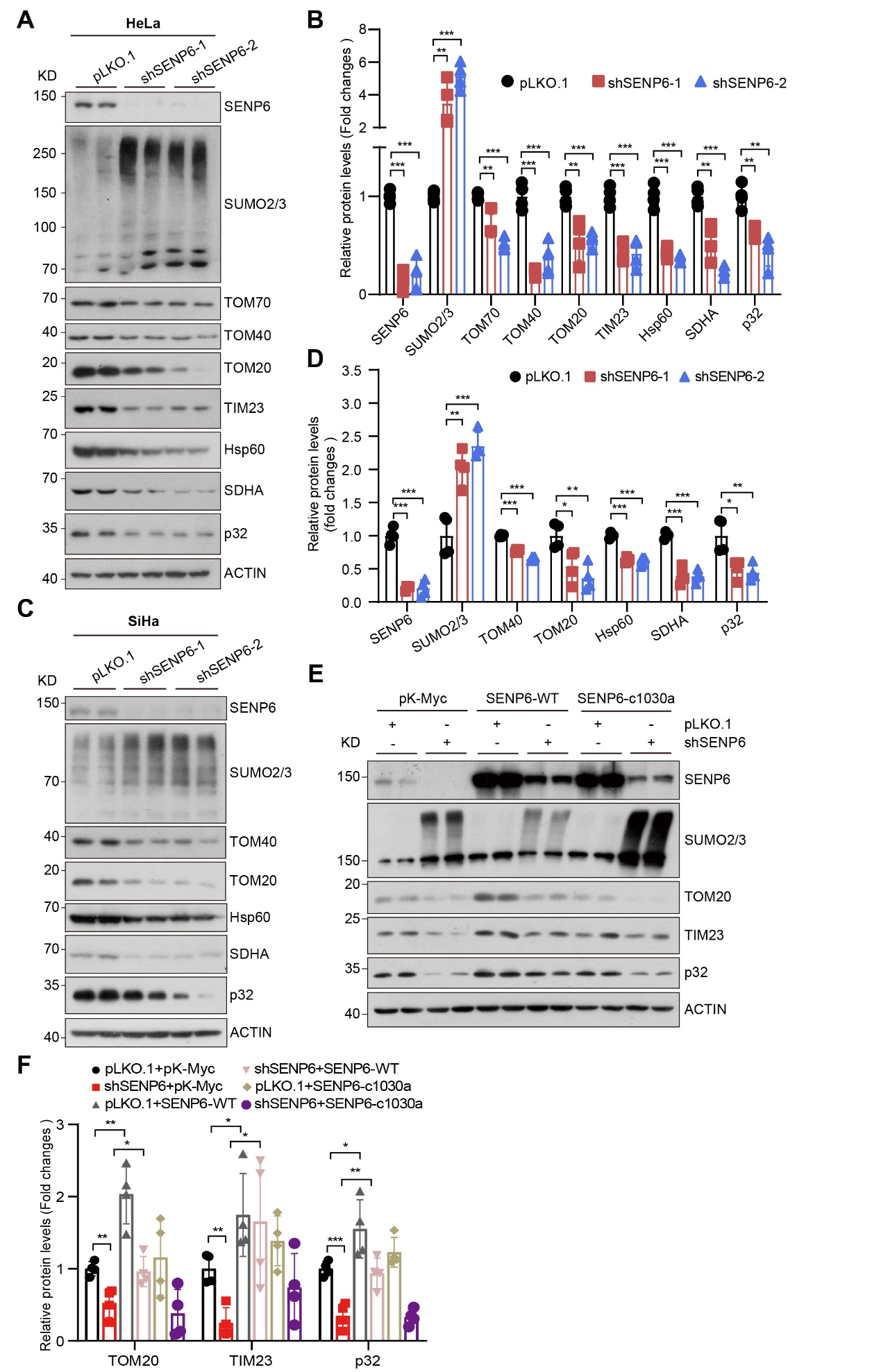


**Figure S2 SENP6 knockdown impairs mitochondrial proteostasis.** (A-B) Western blot analyzed expression of SENP6, SUMO2/3 and mitochondrial-related proteins TOM70, TOM40, TOM20, TIM23, Hsp60, SDHA and p32 in HeLa cells (pLKO.1, shSENP6-1 or shSENP6-2) (n = 4 biologically independent samples; * *P* < 0.05; ** *P* < 0.01; *** *P* < 0.001). (C-D) Western blot analyzed expression of SENP6, SUMO2/3 and mitochondrial-related proteins TOM70, TOM40, TOM20, Hsp60, SDHA and p32 in pLKO.1, shSENP6-1 or shSENP6-2 SiHa cells. (E-F) HeLa cells (pLKO.1 or shSENP6) transiently transfected with FLAG-SENP6-WT or FLAG-SENP6-c1030a, respectively, pK-Myc was used as a control, western blot analyased the expression levels of SENP6, SUMO2/3 and mitochondria-related proteins TOM20, TIM23 and p32 (n = 4 biologically independent samples; * *P* < 0.05; ** *P* < 0.01; *** *P* < 0.001).

**Figure S3**


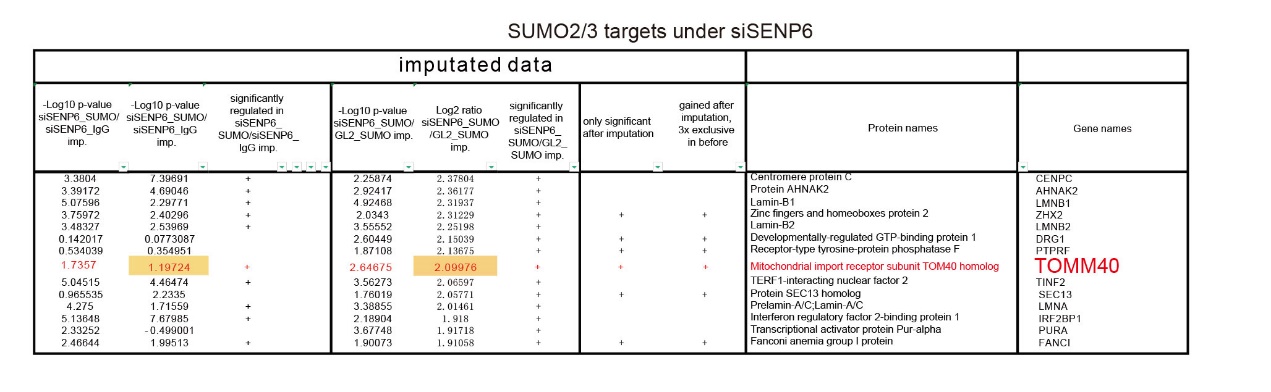


**Figure S3 TOM40 is one of the SUMO2/3 targets in SENP6-depleted HeLa cells.** The dataset of SENP6 target proteins from Dr. Stefan Müller’s published research was subjected to re-analysis.

**Figure S4**

**
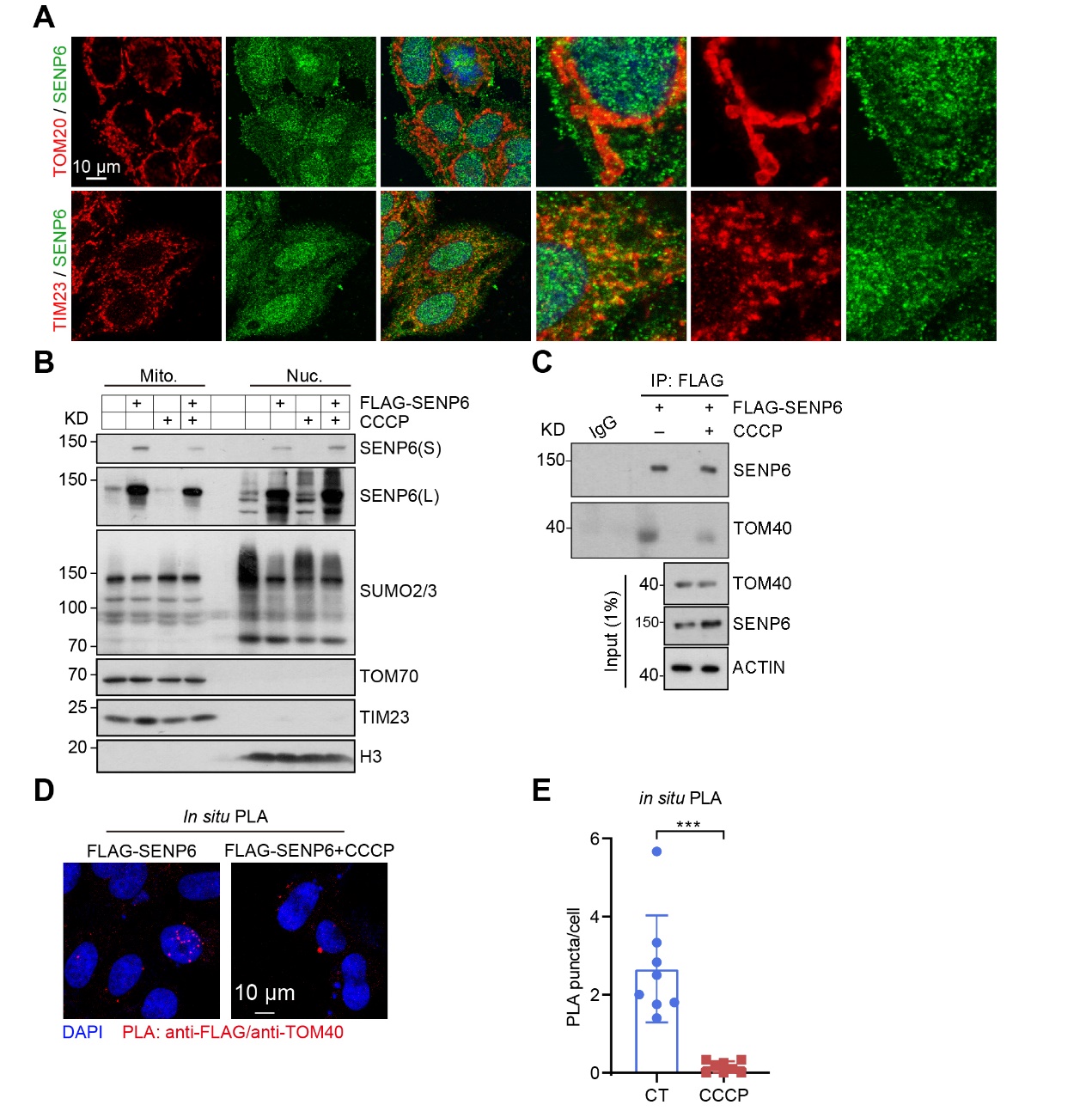
**

**Figure S4. Mitochondria-located SENP6 interacts with TOM40.** (A) Normal HeLa cells were co-stained SENP6 with TOM20 or TIM23. TOM20: mitochondrial outer membrane protein; TIM23: mitochondrial inner membrane protein. Scare bar = 10 μm. (B) Subcellular fraction isolated from HeLa cells was used to detect the expression levels of SENP6, SUMO2/3, TOM70, TIM23 and H3. HeLa cells overexpressed FLAG-SENP6 were treated with DMSO or 10 μM CCCP for 12 hours. (C) 293T cells were transiently transfected with FLAG-SENP6 and treated with DMSO or CCCP (10 μM) for 12 hours. Immunoprecipitation assays were performed with anti-FLAG antibodies to examine the interaction of SENP6 and TOM40. (D-E) Representative images of in situ PLA and quantitative results of PLA puncta per cell. After overexpression of SENP6-FALG, HeLa cells were treated with DMSO or CCCP (10 μM) for 6 hours. (n = 8 biologically independent samples; *** *P* < 0.001; Scale bar, 25 μm).

**Figure S5**

**
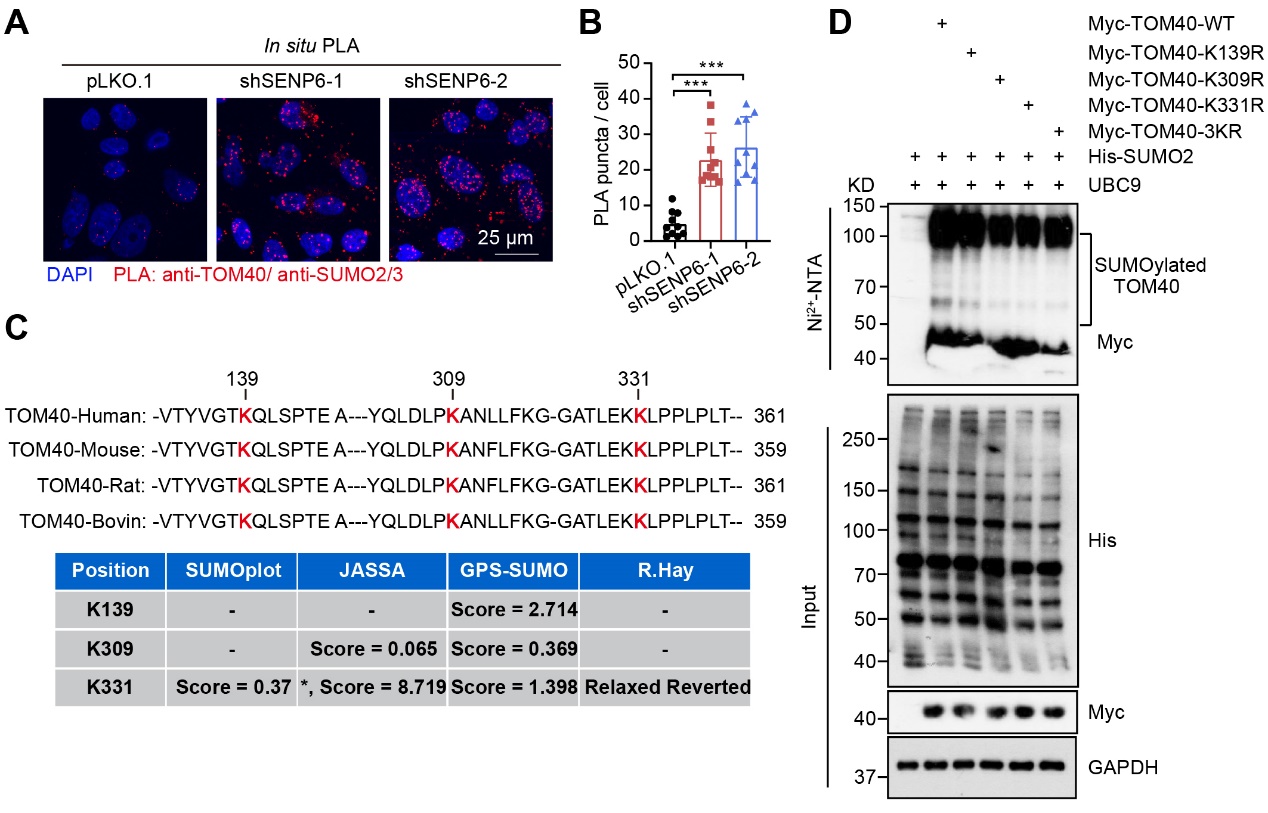
**

**Figure S5. TOM40 is deSUMOylated at K139, K309 and K331 by SENP6.** (A-B) Representative images of *in situ* proximity ligation assay (PLA) in HeLa cells (pLKO.1, shSENP6-1 or shSENP6-2) and quantitative results of PLA puncta per cell. (n = 10 biologically independent samples; *** *P* < 0.001). (C) TOM40 SUMOylation sites were predicted by four prediction tools, including GPS-SUMO, SUMOplot analysis (Abgent) software, JASSA and Ron Hay’s SUMO consensus motif search tool (Bottom). Comparison of the species conservation of TOM40 and SUMOylation modification sites through NCBI (Top). (D) 293T cells were transfected with Ubc9, His-SUMO2, and different mutants of Myc-TOM40 (K139R, K309R, K331R or 3KR). A Ni^2+^-NTA affinity pull-down assay was used for SUMOylation detection.

**Figure S6**

**
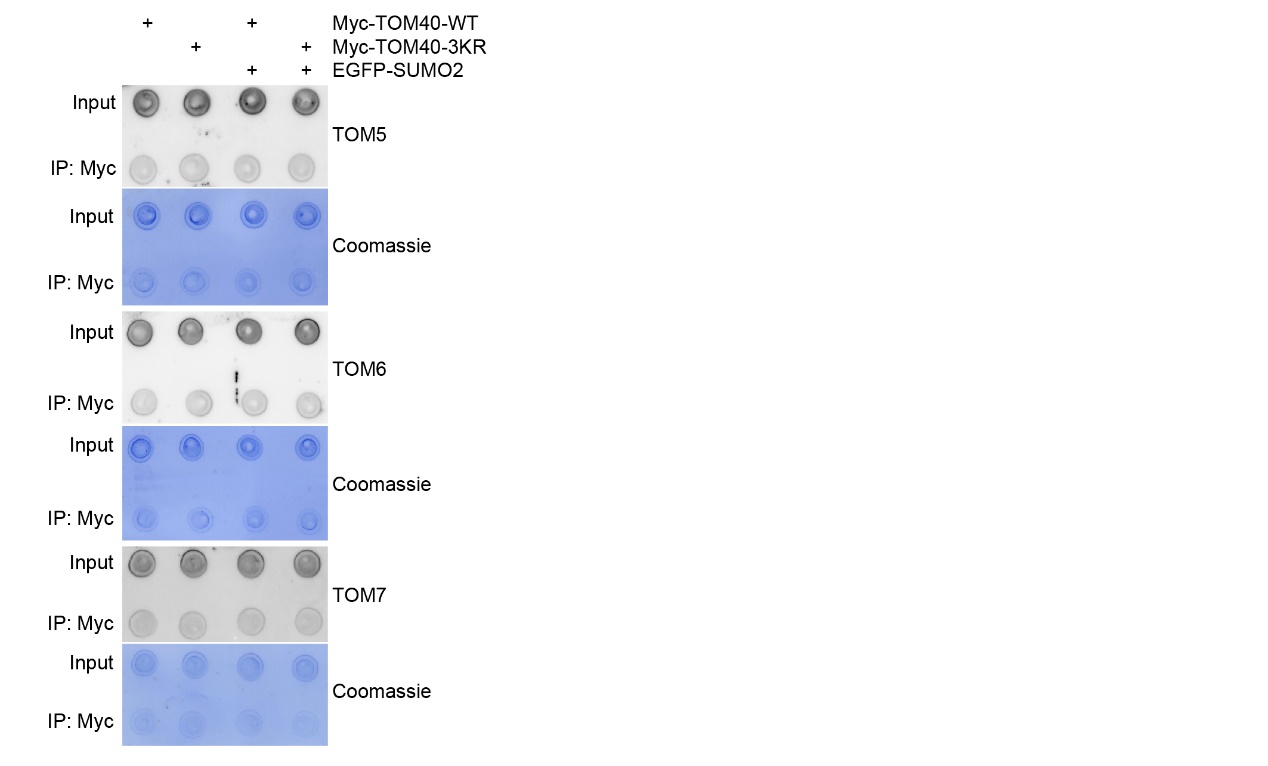
**

**Figure S6. SUMOylation of TOM40 has no effects on the binding with small TOM subunits.** 293T cells were transfected with Myc-TOM40-WT or Myc-TOM40-3KR together with EGFP-SUMO2, or Myc-TOM40-WT, Myc-TOM40-3KR alone for 48 hours. Cell lysis was immunoprecipitated with anti-Myc beads to examine interactions between exogenous Myc-TOM40 and small subunit of TOM complex (TOM5, TOM6 or TOM7). The expression levels of TOM5, TOM6 and TOM7 were analyzed by dot blot.

**Figure S7**

**
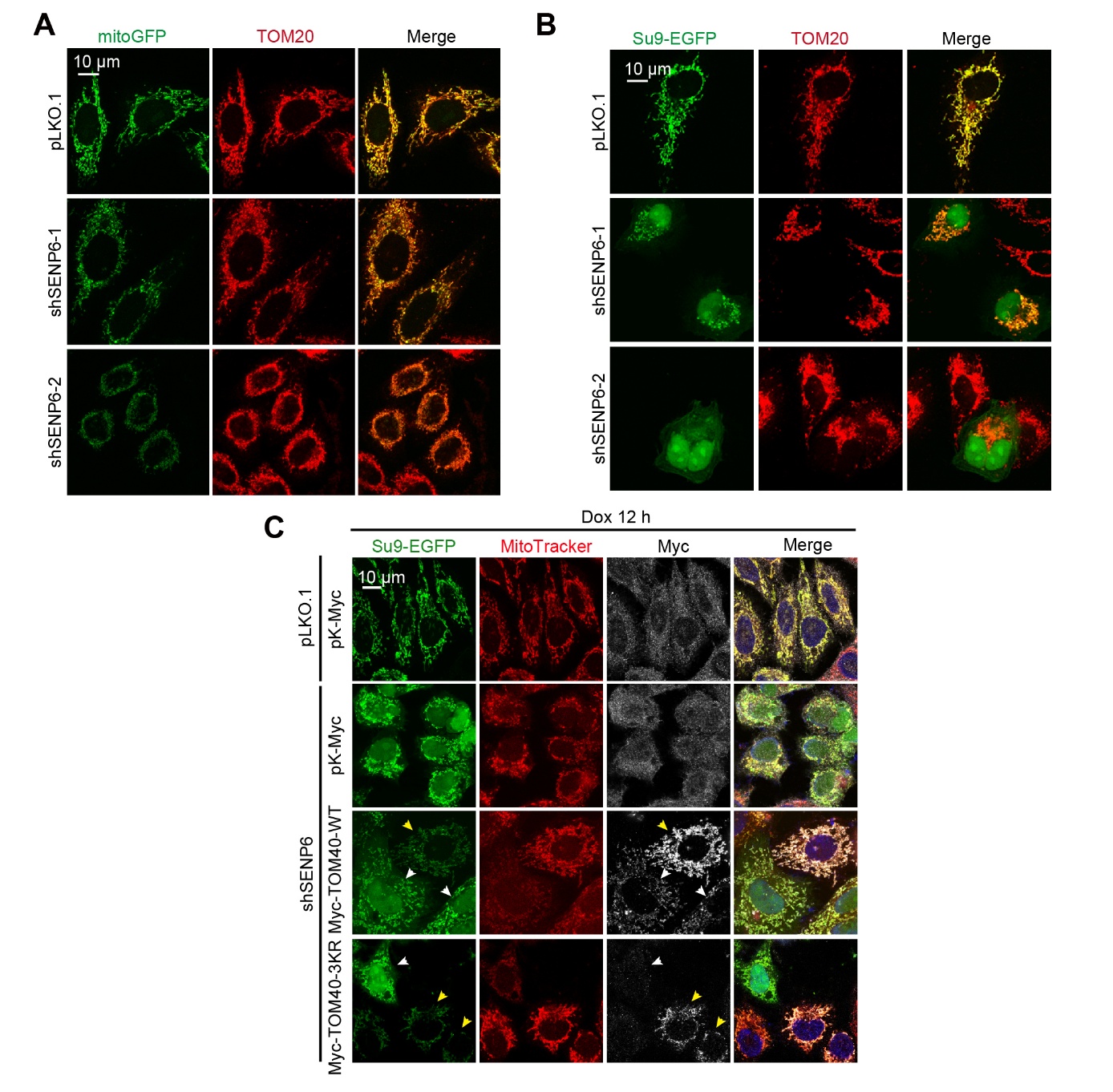
**

**Figure S7. SENP6 knockdown inhibits mitochondrial protein import.** (A) HeLa cells (pLKO.1, shSENP6-1 or shSENP6-2) were transiently transfected with mitoGFP (Green), and anti-TOM20 antibodies were used to label mitochondria (Red). The green fluorescence intensity was observed using a laser confocal microscope. Scale bar, 10 μm. (B) HeLa cells (pLKO.1, shSENP6-1 or shSENP6-2) were transiently transfected with Su9-EGFP (Green), and anti-TOM20 antibodies were used to label mitochondria (Red), the colocalization of green and red fluorescence was observed using a laser confocal microscope. Scale bar, 10 μm. (C) A stable HeLa cell line expressing Dox-Su9-EGFP was established through lentiviral infection and puromycin selection. pK-Myc, Myc-TOM40-WT or Myc-TOM40-3KR were transiently overexpressed in pLKO.1 or shSENP6 Dox-Su9-EGFP-HeLa cells for 48 hours. Expression of Su9-EGFP was induced with Dox for 12 hours. After transfection, cells were labeled with MitoTracke and then stained with Myc antibody. Yellow arrow: cell transfected with Myc-TOM40-WT or Myc-TOM40-3KR; White arrow: cell not transfected, Scare bar = 10 μm.

**Figure S8**

**
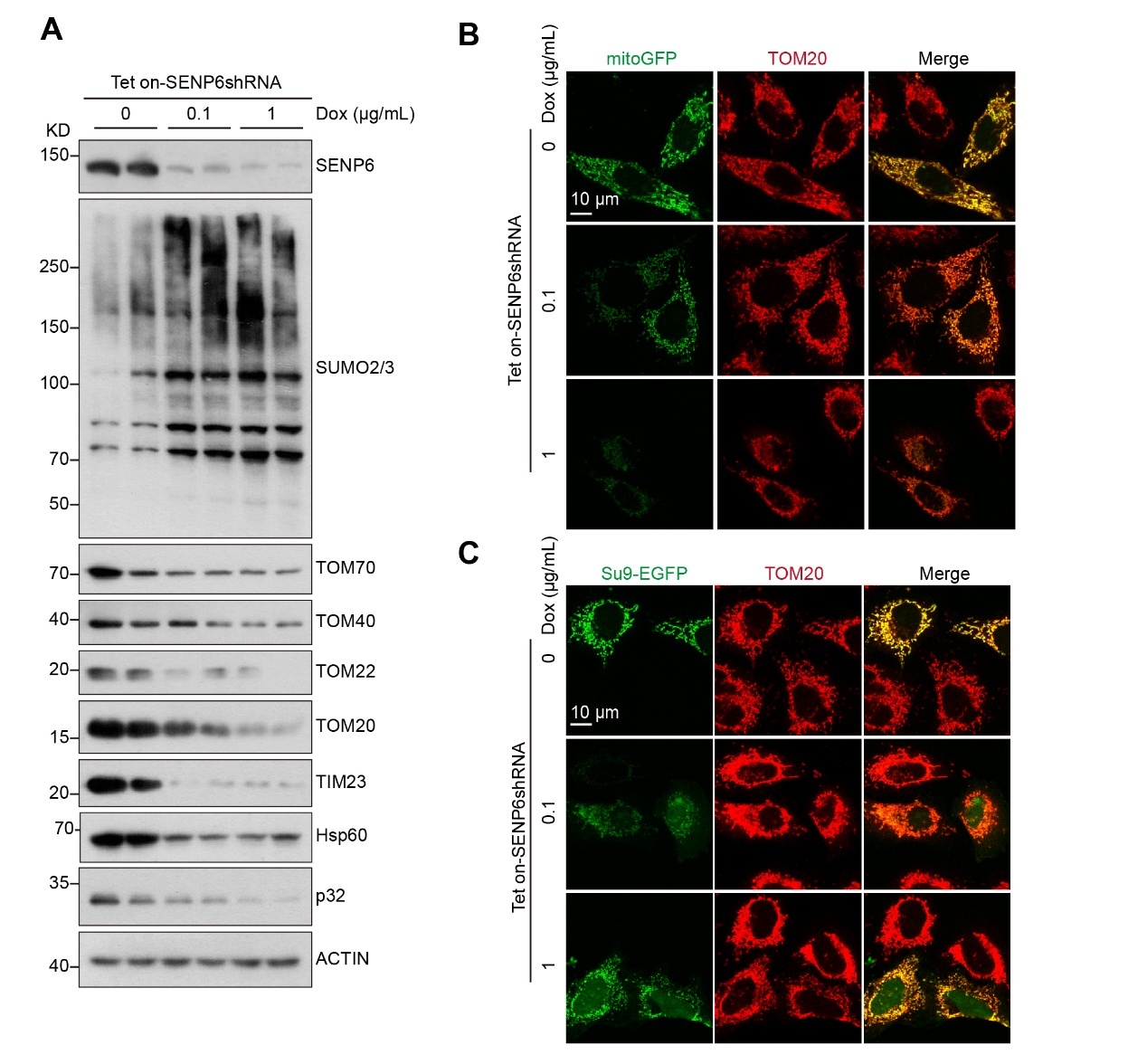
**

**Figure S8. Dox-induced knockdown of SENP6 suppresses mitochondrial protein import.** HeLa cells were induced with different concentrations of Dox (0.1 or 1 μM) to knockdown SENP6. (A) Western blot analysed expression of SENP6, SUMO2/3 and mitochondrial-related proteins TOM70, TOM40, TOM22, TOM20, TIM23, Hsp60 and p32. (B) HeLa cells were transiently transfected with mitoGFP (Green), and anti-TOM20 antibodies were used to label mitochondria (Red). The green fluorescence intensity was observed using a laser confocal microscope. Scale bar, 10 μm. (C) HeLa cells were transiently transfected with Su9-EGFP (Green), and anti-TOM20 antibodies were used to label mitochondria (Red), the colocalization of green and red fluorescence was observed using a laser confocal microscope. Scale bar, 10 μm.

**Figure S9**


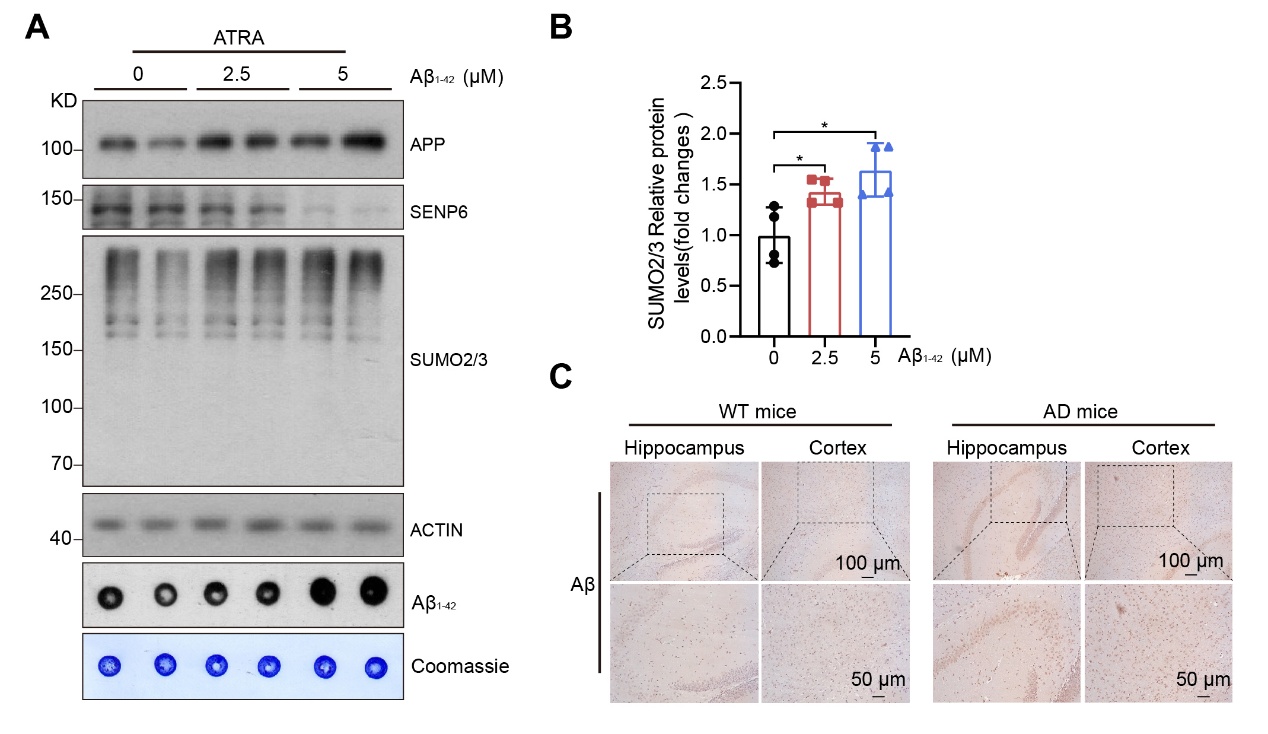


**Figure** **S9. Physiological and pathological relevance and conservation of TOM40 SUMOylation.** (A-B) After treatment with ATRA (10 μM) for 14 days, SH-SY5Y cells were treated with Aβ_1-42_ peptide (0, 2.5 and 5 μM) for 48 hours. Western blot analyzed the expression levels of APP, SENP6 and SUMO2/3, and dot blot analyzed the expression levels of Aβ_1-42_. (n = 4 biologically independent samples; * *P* < 0.05). (C) Immunohistochemistry analyzed the accumulation of Aβ in the hippocampus and cortex of WT and 3×Tg-AD mice (Top scale bar, 100 μm; bottom scale bar, 50 μm).


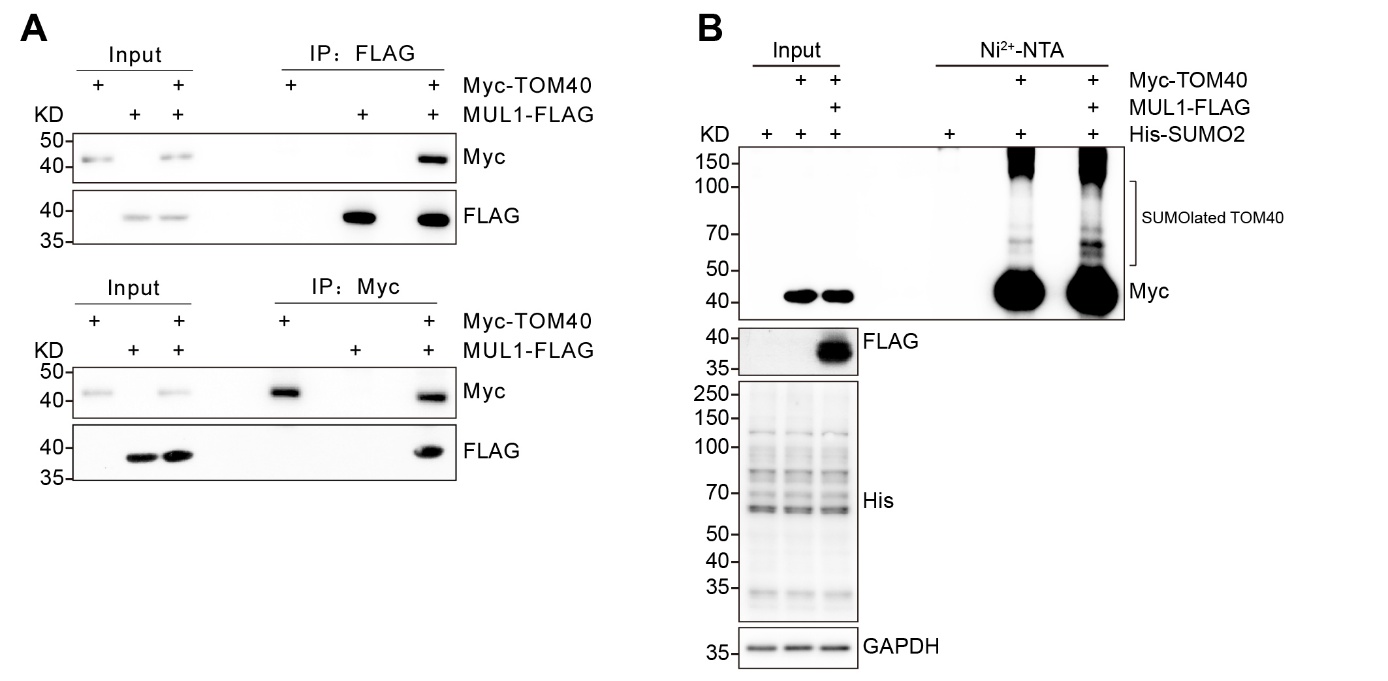


**Figure S10 Mitochondrial SUMO E3 ligase MUL1 improves the SUMOylation of TOM40.** (A) Co-IP analyzed the interaction between MUL1 and TOM40. 293T cells transfected with Myc-TOM40 together with MUL1-FLAG, or Myc-TOM40, MUL1-FLAG alone for 48 hours. Anti-Myc or anti-FLAG beads were used for pulldown. (B) 293T cells were co-transfected with Myc-TOM40 or MUL1-FLAG, together with His-SUMO2. SUMO2 conjugates were pulled down by Ni-NTA affinity isolation.

## Table S1 shRNA sequence

| **Gene** | **Sequence** |
| --- | --- |
| shSENP6-1 | F: 5’-CCGGGAAAGTGACCCTCGTTATAAGCTCGAGCTTATAACGAGGGTCACTTTCTTTTTG-3’ |
|  | R: 5’-AATTCAAAAAGAAAGTGACCCTCGTTATAAGCTCGAGCTTATAACGAGGGTCACTTTC-3’ |
| shSENP6-2 | F: 5’-CCGGGAGTCTACTGGACCATTATTACTCGAGTAATAATGGTCCAGTAGACTCTTTTTG-3’ |
|  | R: 5’-AATTCAAAAAGAGTCTACTGGACCATTATTACTCGAGTAATAATGGTCCAGTAGACTC-3’ |
| shTOM40-1 | F: 5’-CCGGATCACCGCAAGAACAAGTTTCCTCGAGGAAACTTGTTCTTGCGGTGATTTTTTG-3’ |
|  | R: AATTCAAAAAATCACCGCAAGAACAAGTTTCCTCGAGGAAACTTGTTCTTGCGGTGAT-3’ |
| shTOM40-2 | F: 5’-CCGGCATGTCTCTAGCTGGGAAATACTCGAGTATTTCCCAGCTAGAGACATGTTTTTG-3’ |
|  | R: 5’-AATTCAAAAACATGTCTCTAGCTGGGAAATACTCGAGTATTTCCCAGCTAGAGACATG-3’ |

## Table S2 key resources table

| **REAGENT or RESOURCE** | **SOURCE** | | | | **IDENTIFIER** | |  |
| --- | --- | --- | --- | --- | --- | --- | --- |
| **Antibodies** | | | | | | |  |
| anti-Mouse SENP6 | Abnova | | | | Cat: H00026054-M01 | |  |
| anti-Rabbit SDHA | Proteintech | | | | Cat: 14865-1-AP | |  |
| anti-Rabbit TOM70 | Proteintech | | | | Cat: 14528-1-AP | |  |
| anti-Rabbit TOM40 | Proteintech | | | | Cat: 18409-1-AP | |  |
| anti-Rabbit TOM22 | Proteintech | | | | Cat: 11278-1-AP | |  |
| anti-Rabbit TOM20 | Proteintech | | | | Cat: 11802-1-AP | |  |
| anti-Rabbit TIM23 | Proteintech | | | | Cat: 11123-1-AP | |  |
| anti-Rabbit p32 (C1QBP) | Proteintech | | | | Cat: 24474-1-AP | |  |
| anti-Rabbit Hsp60 | Proteintech | | | | Cat: 15282-1-AP | |  |
| anti-Rabbit APP/ Beta Amyloid (Aβ) | Proteintech | | | | Cat: 25524-1-AP | |  |
| anti-beta Amyloid (MOAB-2) | Novus Biologicals | | | | Cat: NBP2-13075 | |  |
| anti-Rabbit Myc-tag | Proteintech | | | | Cat: 16286-1-AP | |  |
| anti-Mouse Myc-tag | Proteintech | | | | Cat: 60003-2-Ig | |  |
| anti-Rabbit SUMO2/3（R） | Proteintech | | | | Cat: 11251-1-AP | |  |
| anti-Rabbit SUMO2/3（M） | Millipore | | | | Cat: MABS2039 | |  |
| anti-Mouse β-actin | Sigma Aldrich | | | | Cat: A1978, AC-15 | |  |
| anti-Rabbit H3 | Cell Signaling Technology | | | | Cat: 9715 | |  |
| anti-Mouse-His | Proteintech | | | | Cat: 66005-1-Ig | |  |
| TOM5 | Proteintech | | | | Cat: 25607-1-AP | |  |
| TOM6 | Proteintech | | | | Cat: 16689-1-AP | |  |
| TOM7 | Proteintech | | | | Cat: 15071-1-AP | |  |
| Normal IgG (Rabbit) | Cell Signaling Technology | | | | Cat: 7074 | |  |
| Normal IgG (Mouse) | Santa Cruz Biotechnology | | | | Cat: sc-2025 | |  |
| VeriBlot for IP Detection | abcam | | | | Cat: ab131366 | |  |
| Alexa Fluor® 488-AffiniPure Goat Anti-Rabbit | Jackson Immunoresearch | | | | Cat: 111-545-144 | |  |
| Alexa Fluor® 594-AffiniPure Goat Anti-Rabbit | Jackson Immunoresearch | | | | Cat: 115-585-146 | |  |
| Alexa Fluor® 488-AffiniPure Goat Anti-Mouse | Jackson Immunoresearch | | | | Cat: 115-545-146 | |  |
| **Chemicals, peptides, and recombinant proteins** | | | | | | |  |
| Doxycycline Hydrochloride | Selleck | | | | Cat: S4163 | |  |
| CCCP | MedChemExpress | | | | Cat: HY-100941 | |  |
| Digitonin | Sigma Aldrich | | | | Cat: D141 | |  |
| NEM | Sigma Aldrich | | | | Cat: E3876 | |  |
| Aβ peptide (1-42) human | Beyotime Biotechnology | | | | Cat: P9001 | |  |
| ATRA (Retinoic acid) | Beyotime Biotechnology | | | | Cat: ST1627 | |  |
| PMSF | Beyotime Biotechnology | | | | Cat: ST506 | |  |
| Protease inhibitor cocktail | Bimake | | | | Cat: B14002 | |  |
| Puromycin Dihydrochloride | Amresco | | | | Cat: J539 | |  |
| Protein A/G Magnetic beads | MedChemExpress | | | | Cat: HY-K0202 | |  |
| M10×Blue Native Transfer Buffer | Real-Times Biotechnology | | | | Cat: BC600P | |  |
| RealPAGE Native BN/CN Precast Gels | | | Real-Times Biotechnology | Cat: RTD6138-0416 | | | |
| Brilliant Blue G | Beyotime Biotechnology | | | | ST1119 | |  |
| 10×BN/CN PAGE Running Buffer | Real-Times Biotechnology | | | | Cat: BC500P | |  |
| FastPfu Fly DNA Polymerase | TransGen Biotech | | | | Cat: AP231-01 | |  |
| T4 DNA ligase | Takara | | | | Cat: 2011A | |  |
| PEI | Polysciences Inc | | | | Cat: 24765-2 | |  |
| Opti-MEM | Gibco | | | | Cat: 2003808 | |  |
| DAPI  MitoTracker Red | Sigma Aldrich  Invitrogen | | | | Cat: D8417  Cat.:M7513 | |  |
| Ni-NTA Agarose | QIAGENE | | | | Cat: 30210 | |  |
| **Critical commercial assays** | | | | | | |  |
| Seahorse XF Cell Mito Stress Test Kit | Agilent | | | | Cat: 103015-100 | |  |
| Duolink^®^ In Situ Red Starter Kit Mouse/ Rabbit | Sigma Aldrich | | | | Cat: DUO92101 | |  |
| ROS Assay Kit | Beyotime Biotechnology | | | | Cat: S0033S | |  |
| MitoSOX^TM^ Red mitochondrial superoxide indicator | ThermoFisher Scientific | | | | Cat: M36008 | |  |
| Mitochondrial membrane potential assay kit with JC-1 | Beyotime Biotechnology | | | | Cat: C2006 | |  |
| SUMOylation kit | Enzo Life Sciences | | | | Cat: BML-UW8955-0001 | |  |
| Anti-Myc MagBeads | | Yeasen Biotechnology | | | | Cat: 20567ES03 | |
| Anti-Flag NanoMagBeads | | Fitgene | | | | Cat: FI8201 | |
| **Experimental models: Cell lines** | | | | | | |  |
| Human cervical cancer cell HeLa | Shanghai Cell Bank | | | | SCSP-504 | |  |
| Human cervical cancer cell SiHa | Shanghai Cell Bank | | | | SCSP-5058 | |  |
| Human embryonic kidney cell line 293T | Shanghai Cell Bank | | | | SCSP-502 | |  |
| Human Neuroblastoma Cell Line (SH-SY5Y) | Shanghai Cell Bank | | | | SCSP-5014 | |  |
| C57BL/6 3×Tg-AD | Aniphe Biolaboratory | | | | DM11003 | |  |
| **Oligonucleotides** | | | | | | |  |
| shRNA sequences, see Table S1 | This paper | | | | N/A | |  |
| **Recombinant DNA** | | | | | | |  |
| Myc-TOM40-WT | This paper | | | | N/A | |  |
| Myc-TOM40-K139R | This paper | | | | N/A | |  |
| Myc-TOM40-K309R | This paper | | | | N/A | |  |
| Myc-TOM40-K331R | This paper | | | | N/A | |  |
| Myc-TOM40-3KR | This paper | | | | N/A | |  |
| FLAG-SENP6-WT | Addgene | | | | Cat: 18065 | |  |
| FLAG-SENP6-c1030a | Addgene | | | | Cat: 18716 | |  |
| EGFP-SUMO2 | This paper | | | | N/A | |  |
| HA-SUMO2 | This paper | | | | N/A | |  |
| His-SUMO2 | Miaoling Biotechnology | | | | Cat: P57217; http://www.miaolingbio.com/plasmid/P57217.html#zlxl | |  |
| pUBC9 | This paper | | | | N/A | |  |
| Su9-EGFP | Addgene | | | | Cat: 23214 | |  |
| Dox-Su9-EGFP | This paper | | | | N/A | |  |
| Dox-Su9-DHFR-3×FLAG | This paper | | | | N/A | |  |
| pLV[Exp]-CMV>{MTS_mScarlet-GFP1-10}:IRES:Puro | Vectorbuilder | | | | https://www.vectorbuilder.cn/vector/VB240304-1158qjv.html | |  |
| pRP[TetOn]-TRE>XhoI/hNDUFA4/BglII:GSlinker:GFP11 | Vectorbuilder | | | | https://www.vectorbuilder.cn/vector/VB240304-1660qgg.html | |  |
| mitoGFP | Gift from Song Lab Wuhan University | | | | N/A | |  |
| **Software and Algorithms** | | | | | | |  |
| GraphPad Prism 8 | https://www.graphpad.com/ | | | | N/A | |  |
| Image J | https://imagej.nih.gov/ij/ | | | | N/A | |  |
| Quantity One | https://www.bio-rad.com/ | | | | N/A | |  |
