# Supplementary material for: SENP6 Maintains Mitochondrial Homeostasis by Regulating Mitochondrial Protein Import Through deSUMOylation of TOM40
Source: Adv Sci (Weinh). 2025 Jul 29;12(40):e03408. doi: 10.1002/advs.202503408 (PMC12561365; doi:10.1002/advs.202503408)
Supplement: Supplementary file 2 — Supporting Information [file ADVS-12-e03408-s001.docx]

**Figure 1**

**
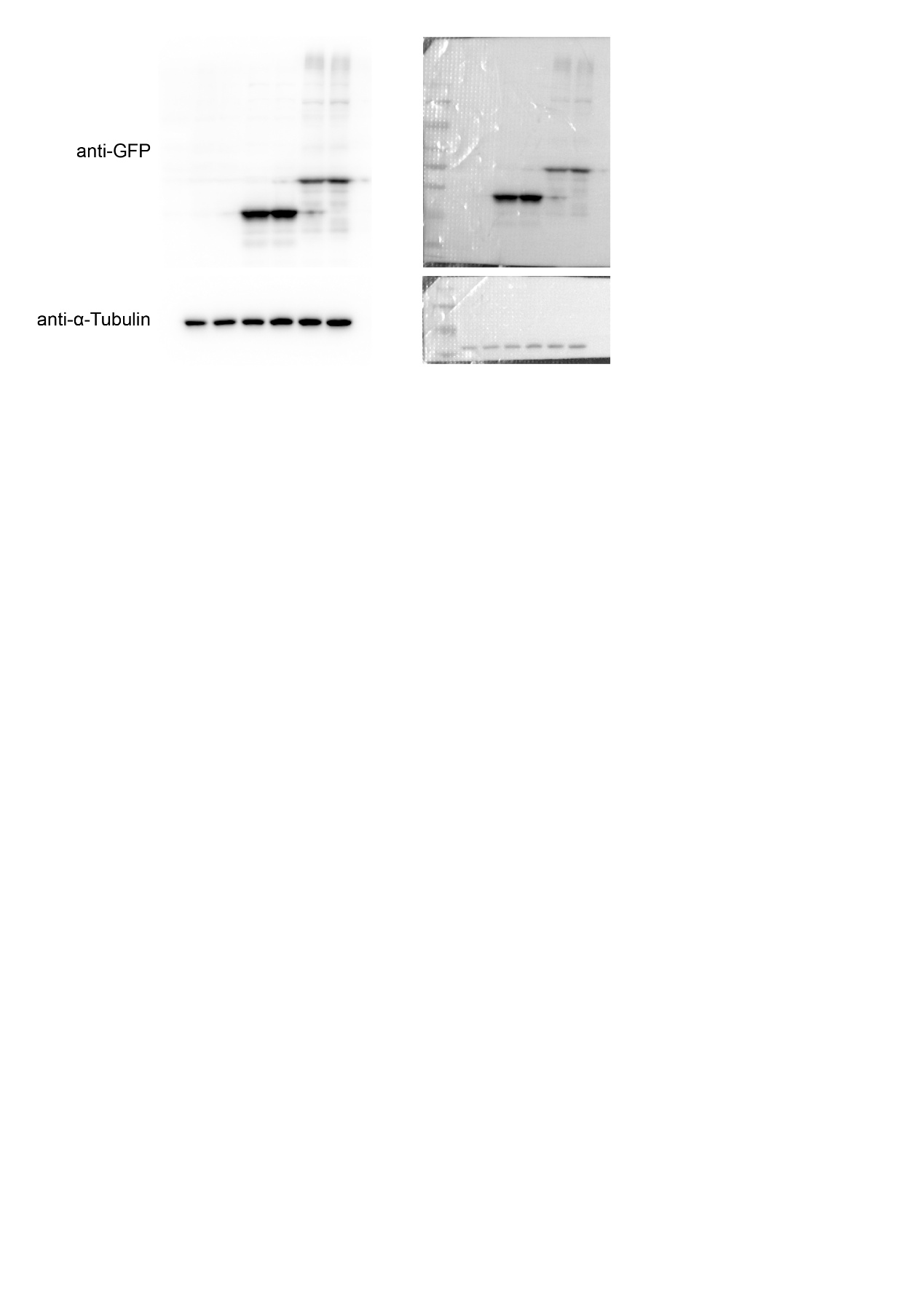
**

**Figure 2**

**
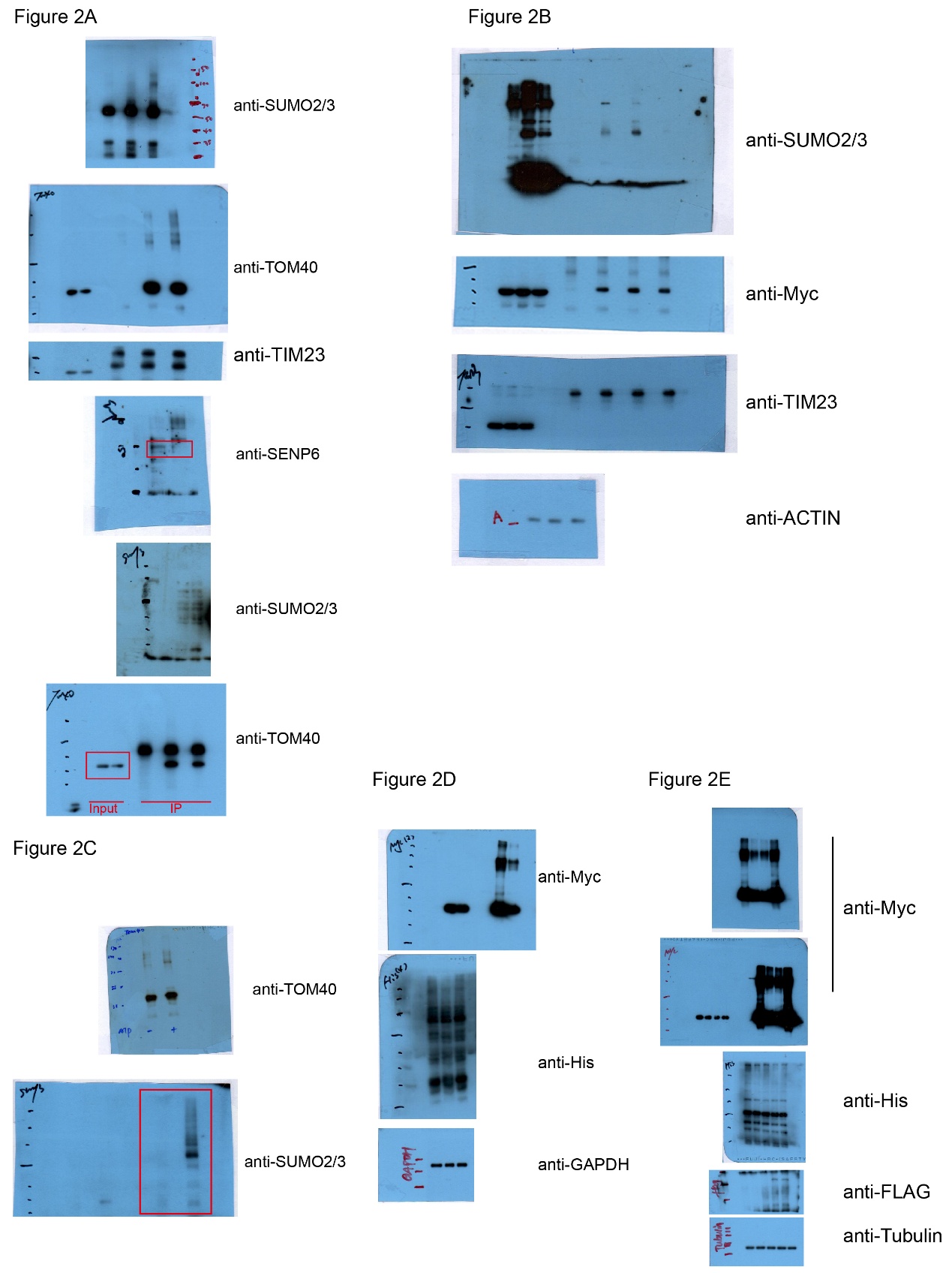
**

**Figure 3**

**
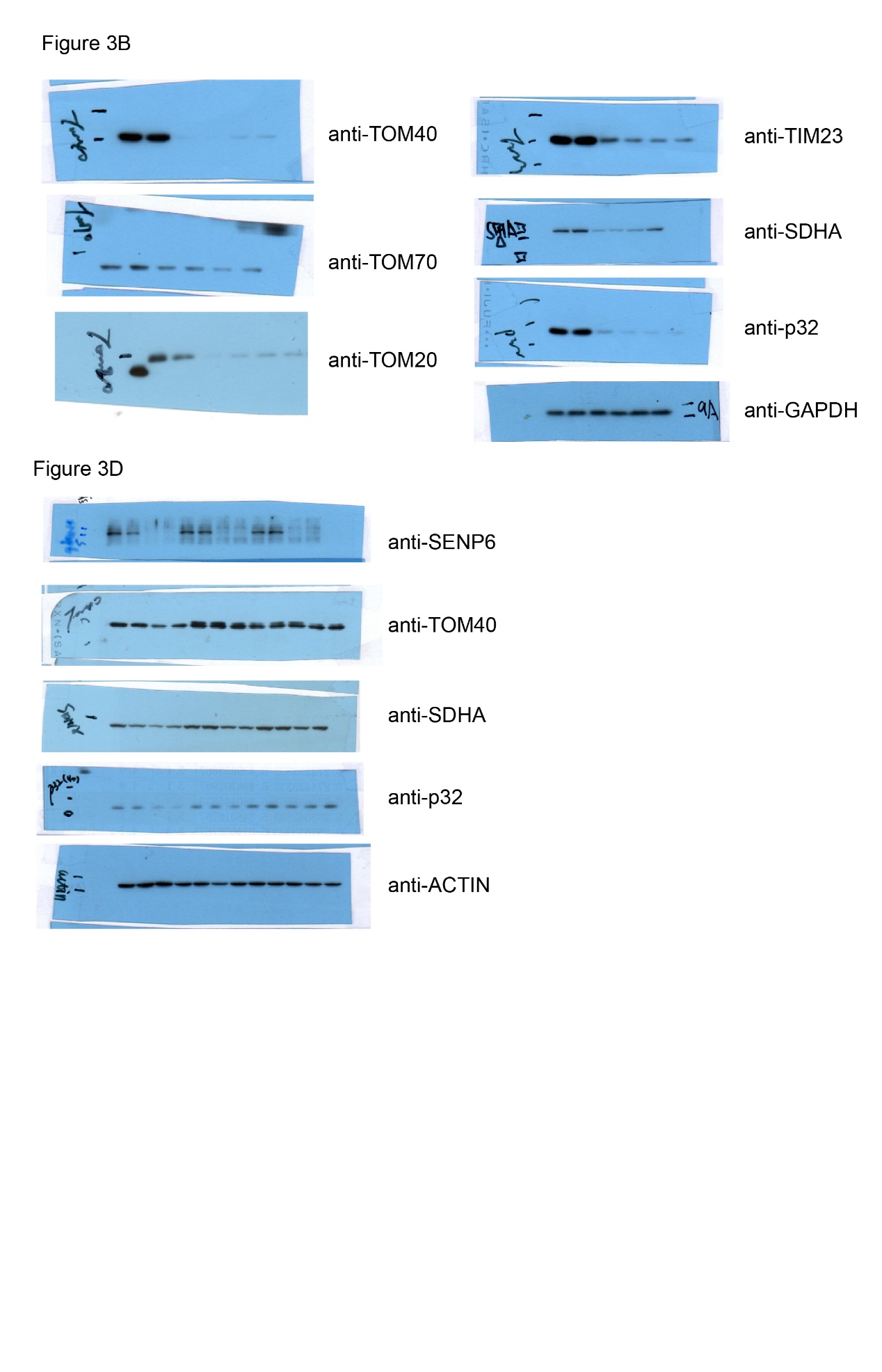
**

**Figure 4**

**
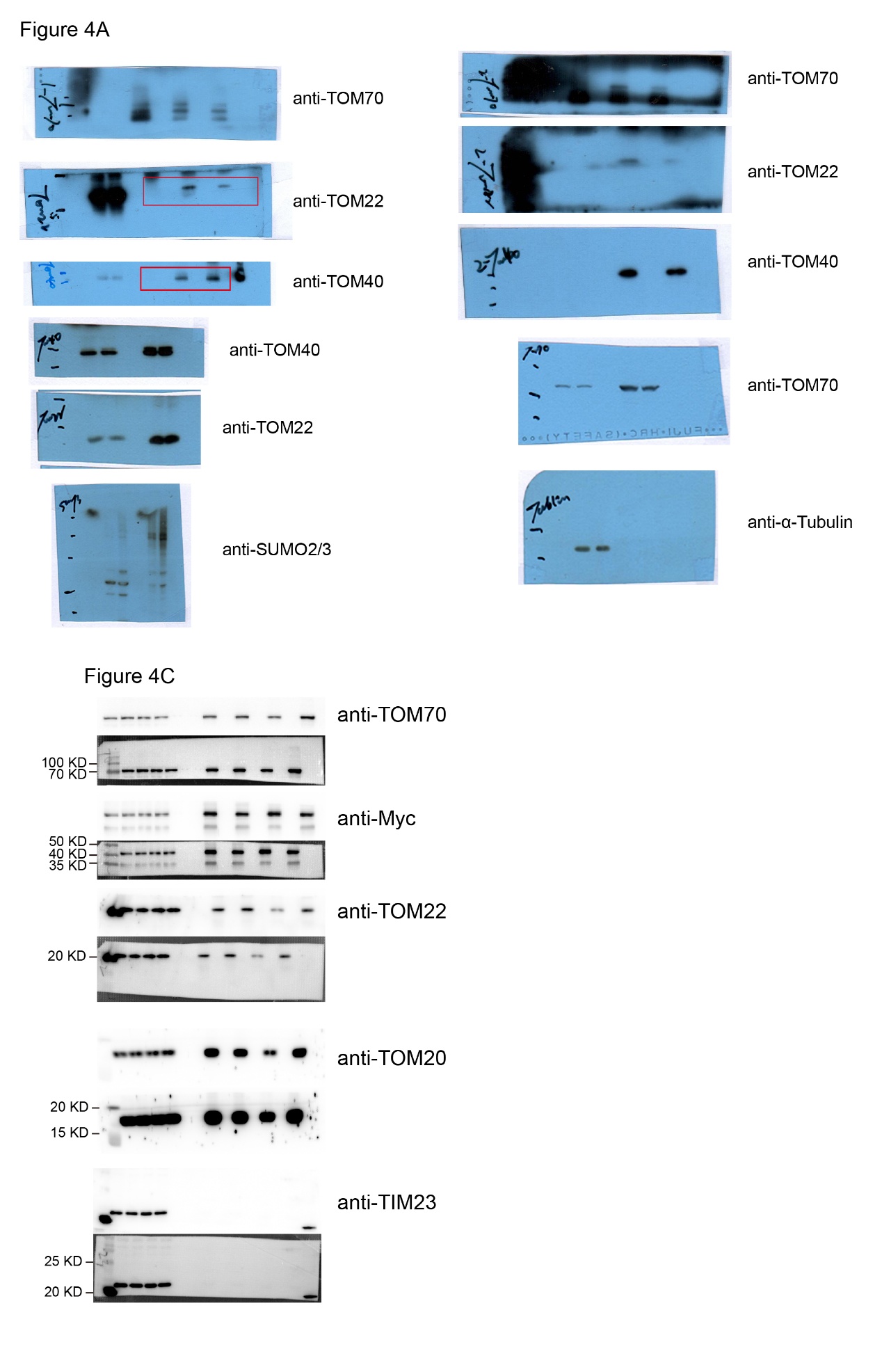
**

**
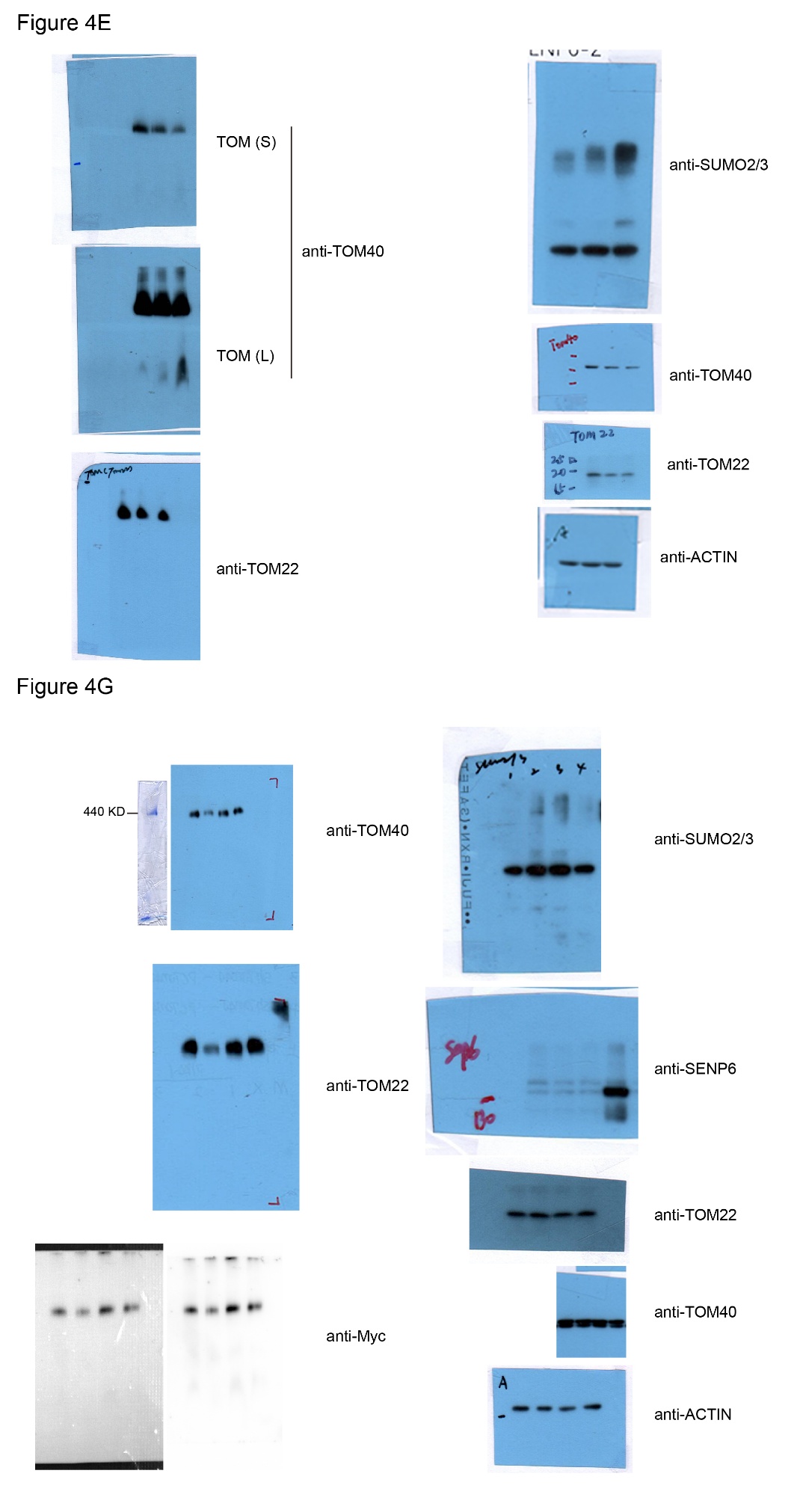
**

**Figure 5**

**
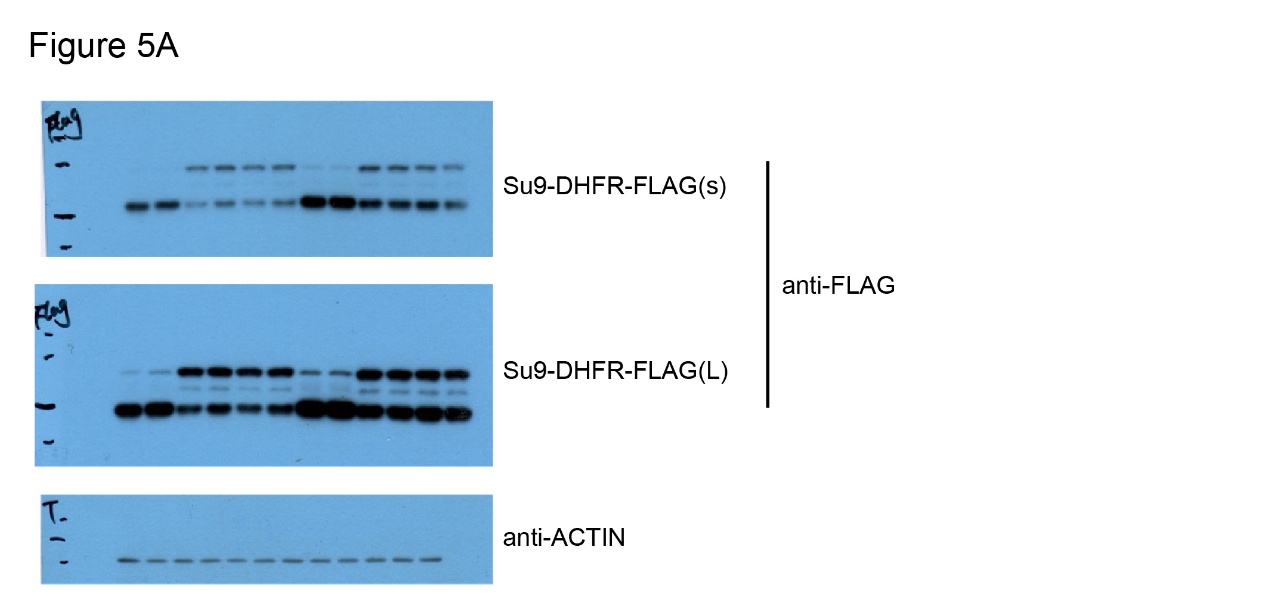
**

**Figure 6**

**
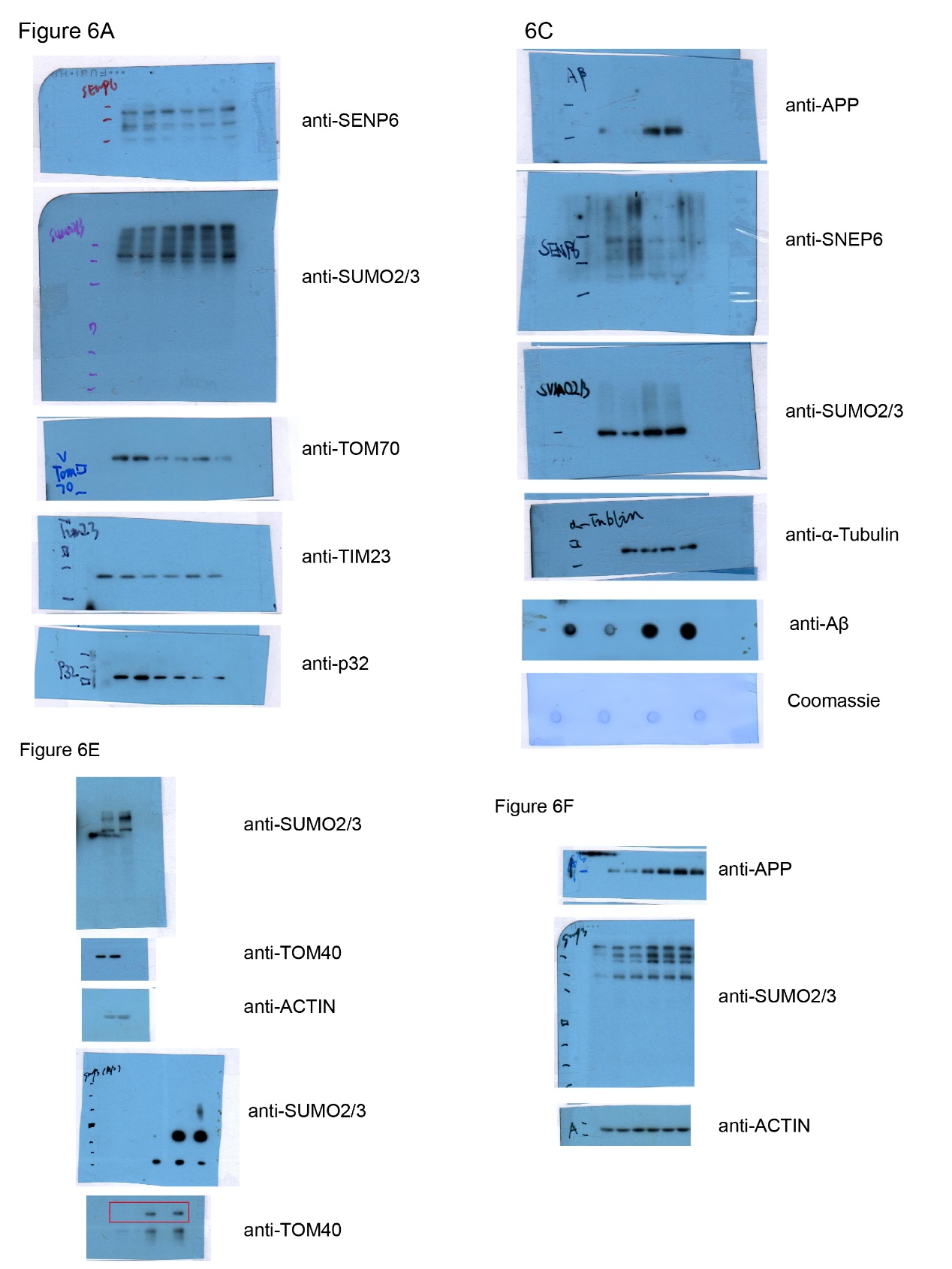
**

**
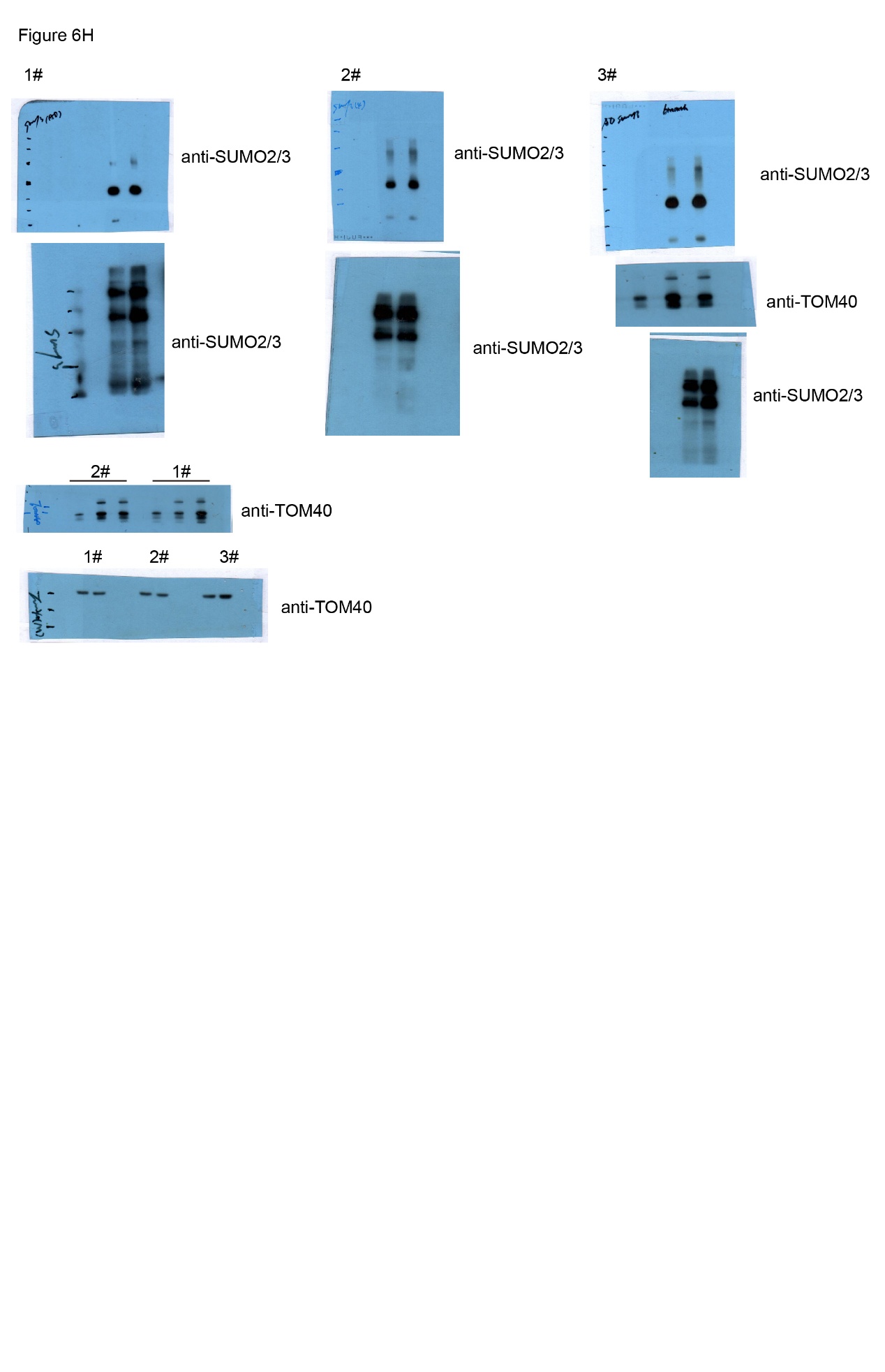
**

**Figure S2**

**
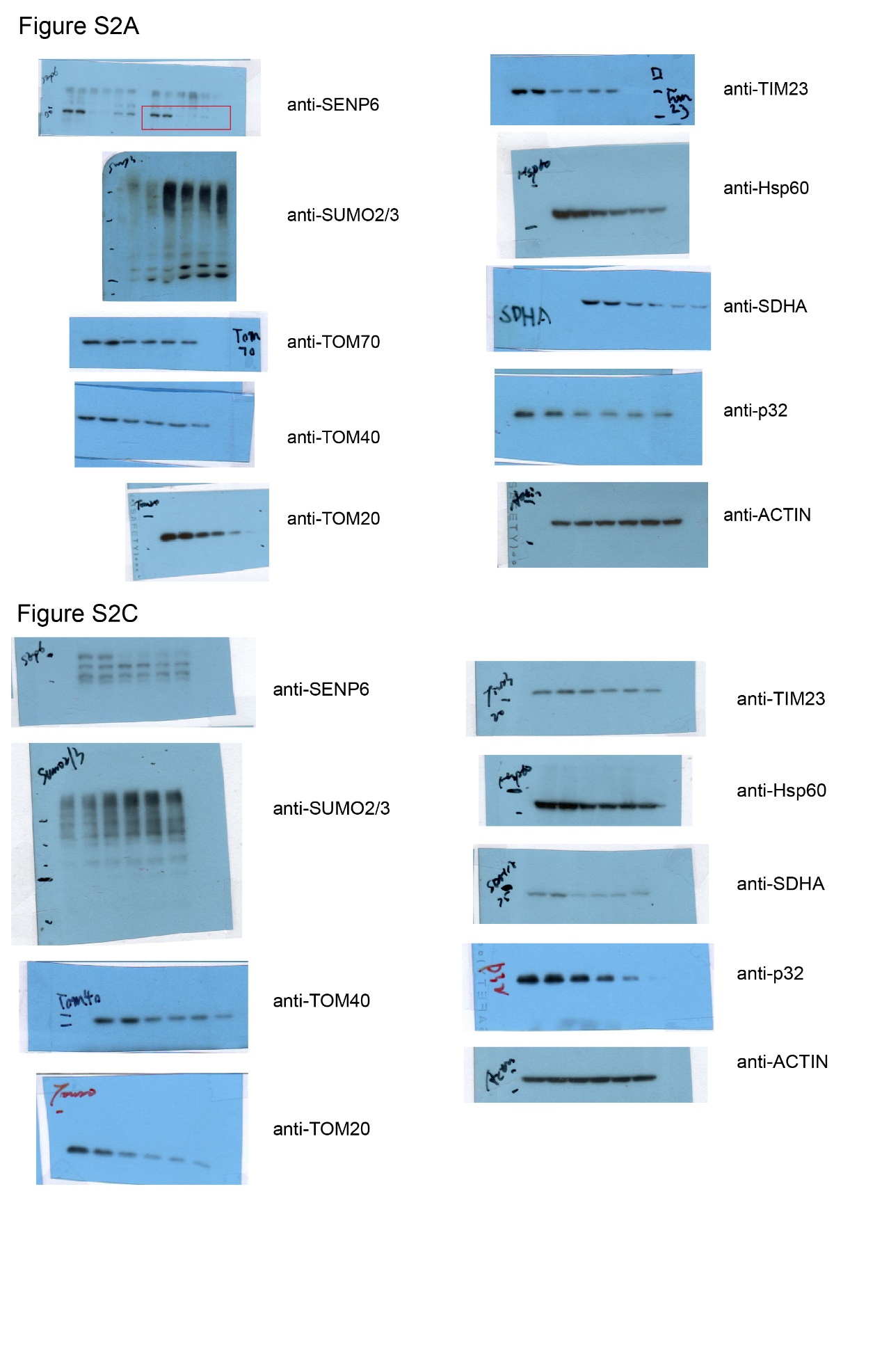
**

**
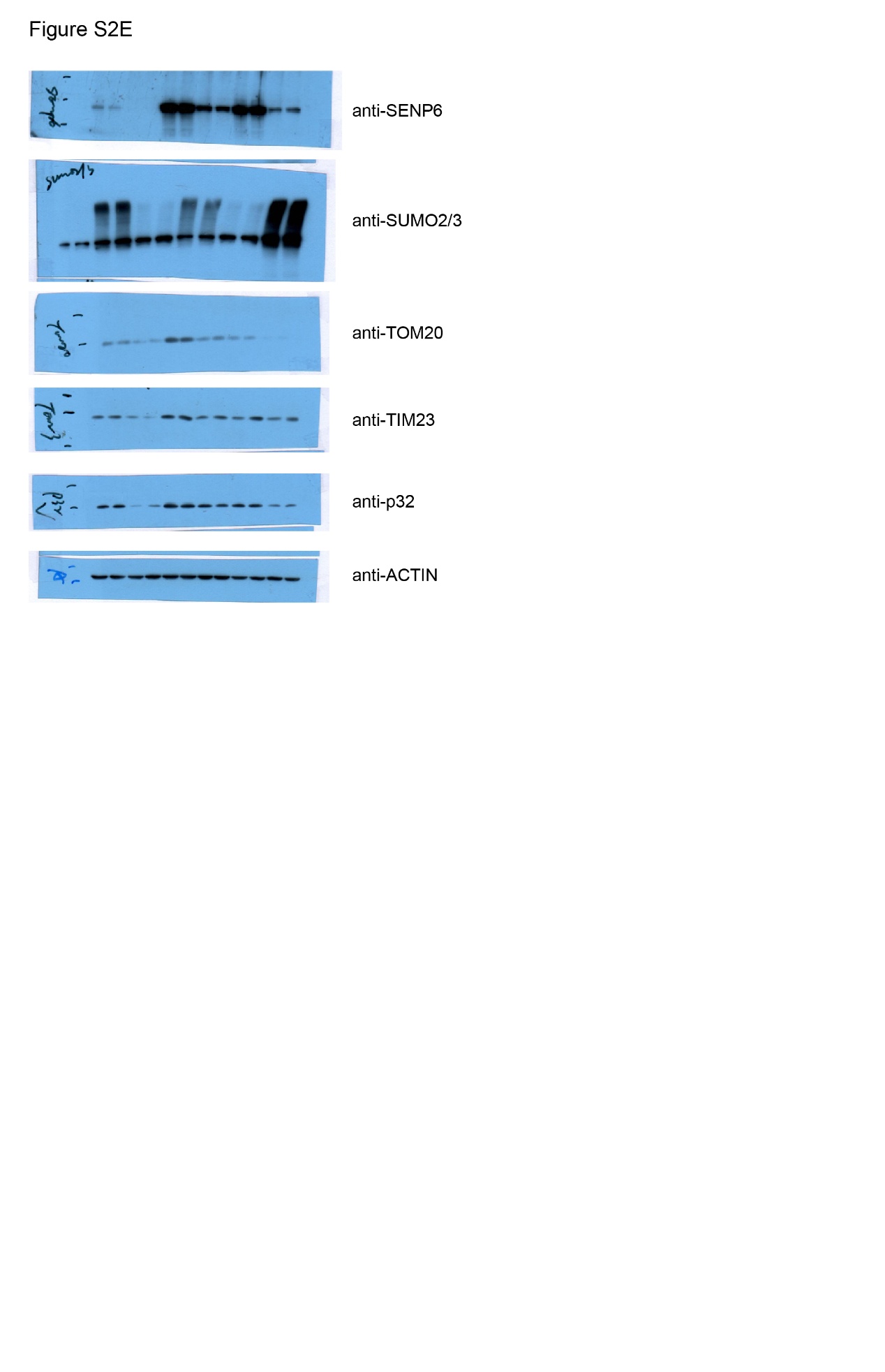
**

**Figure S4**

**
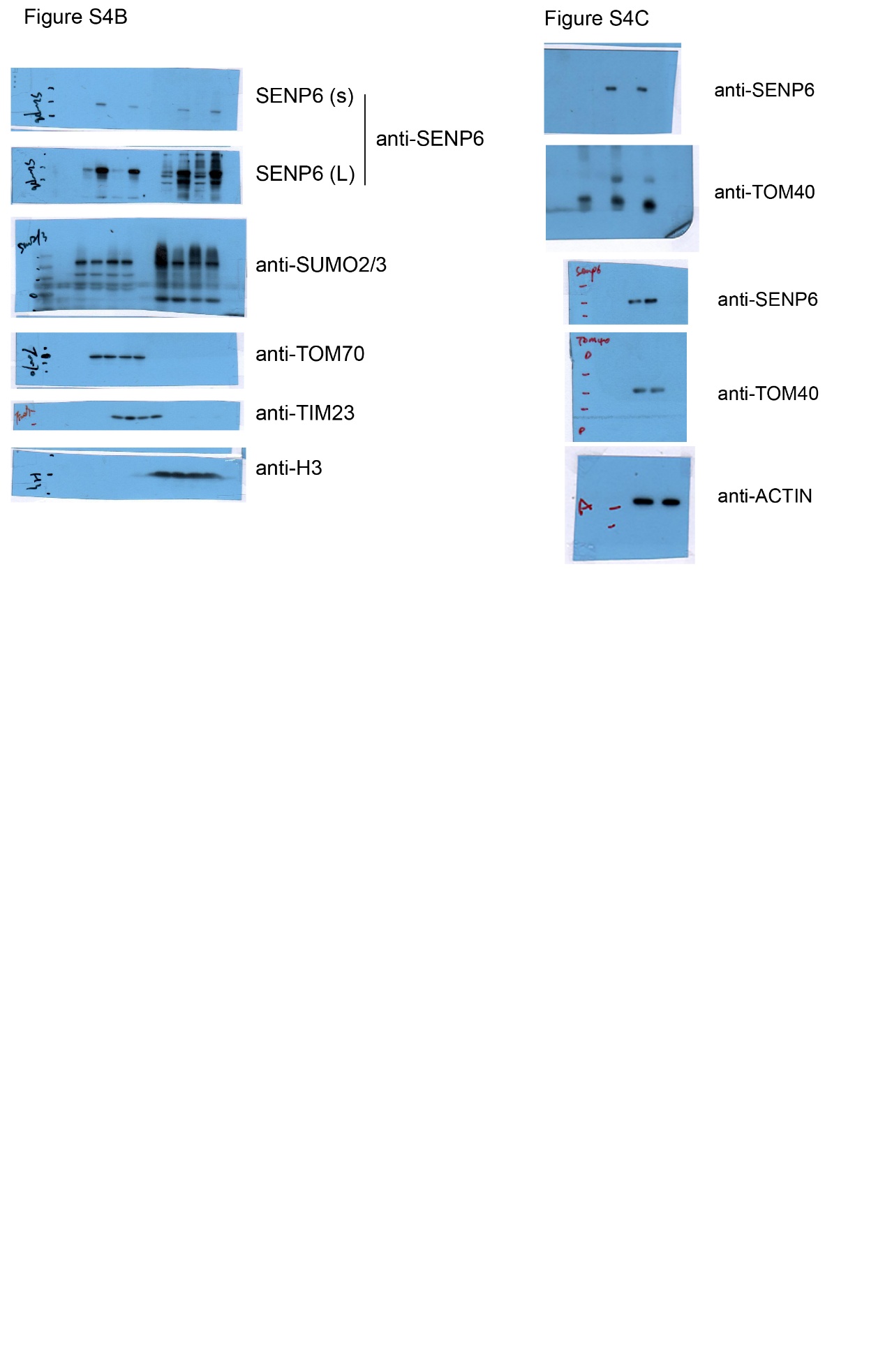
**

**Figure S5**

**
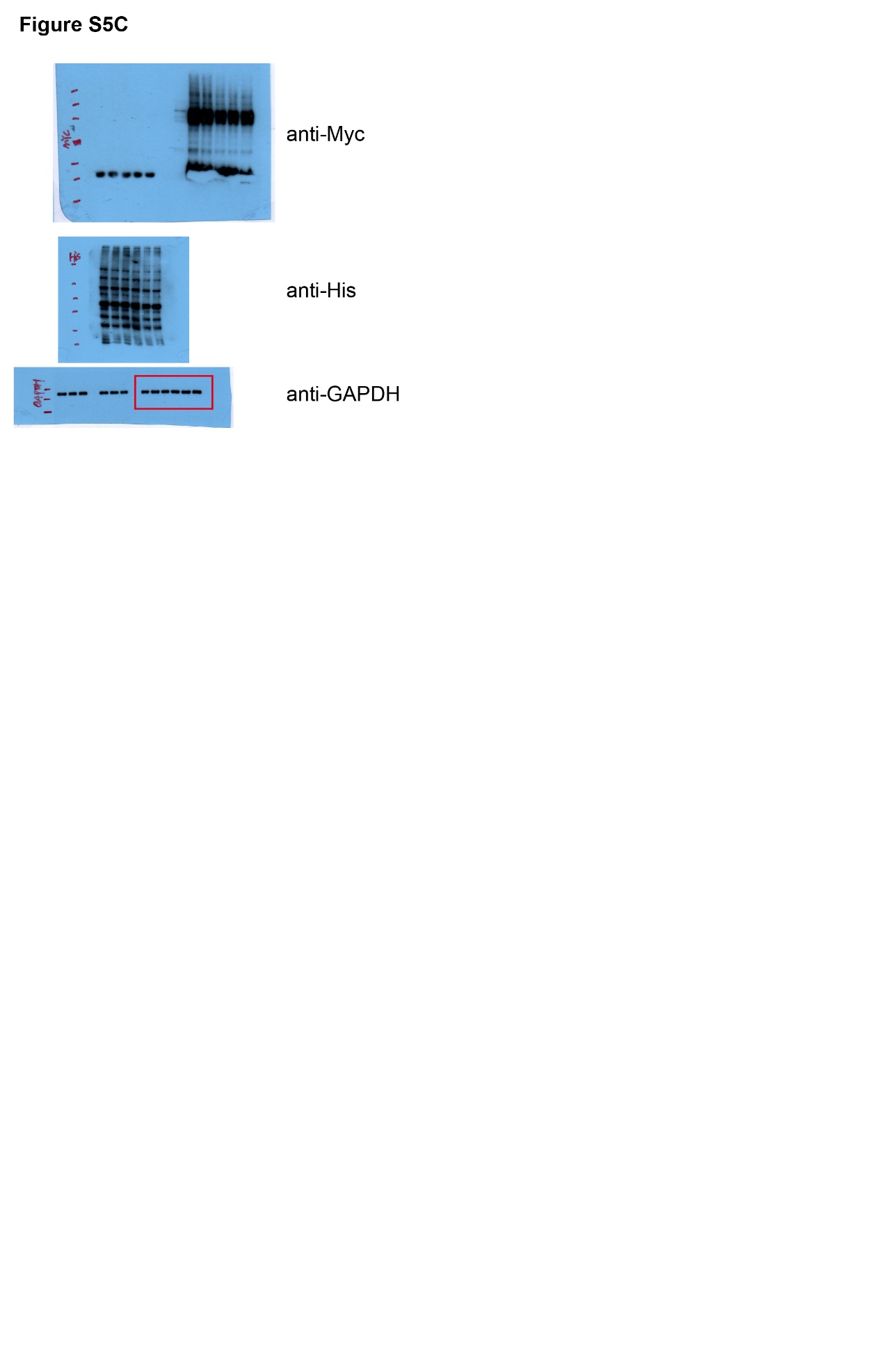
**

**Figure S6**

**
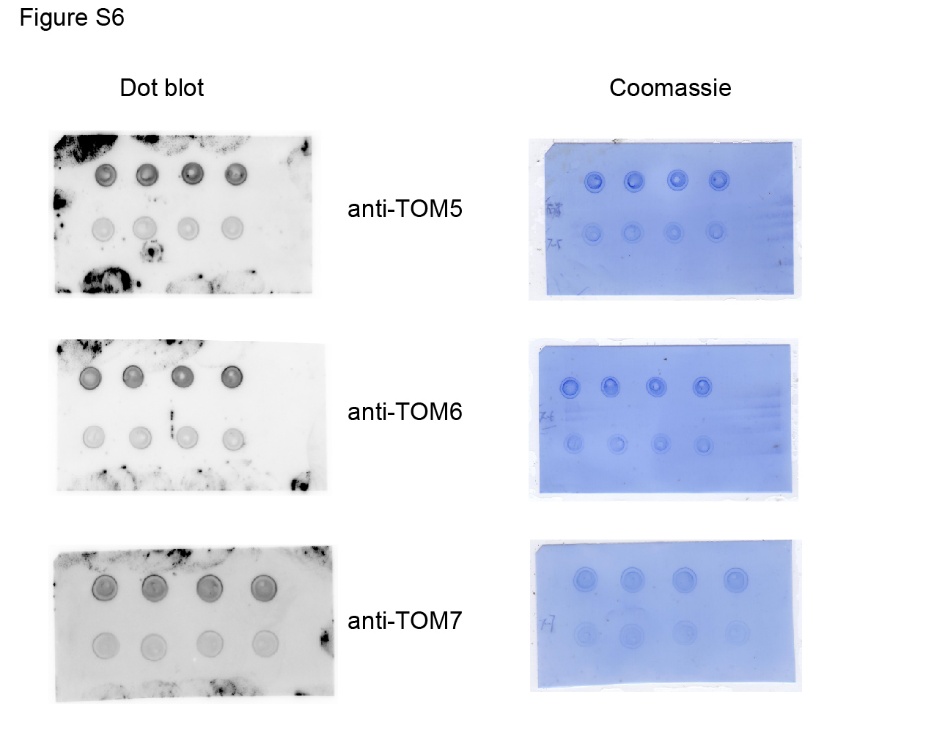
**

**Figure S8**

**
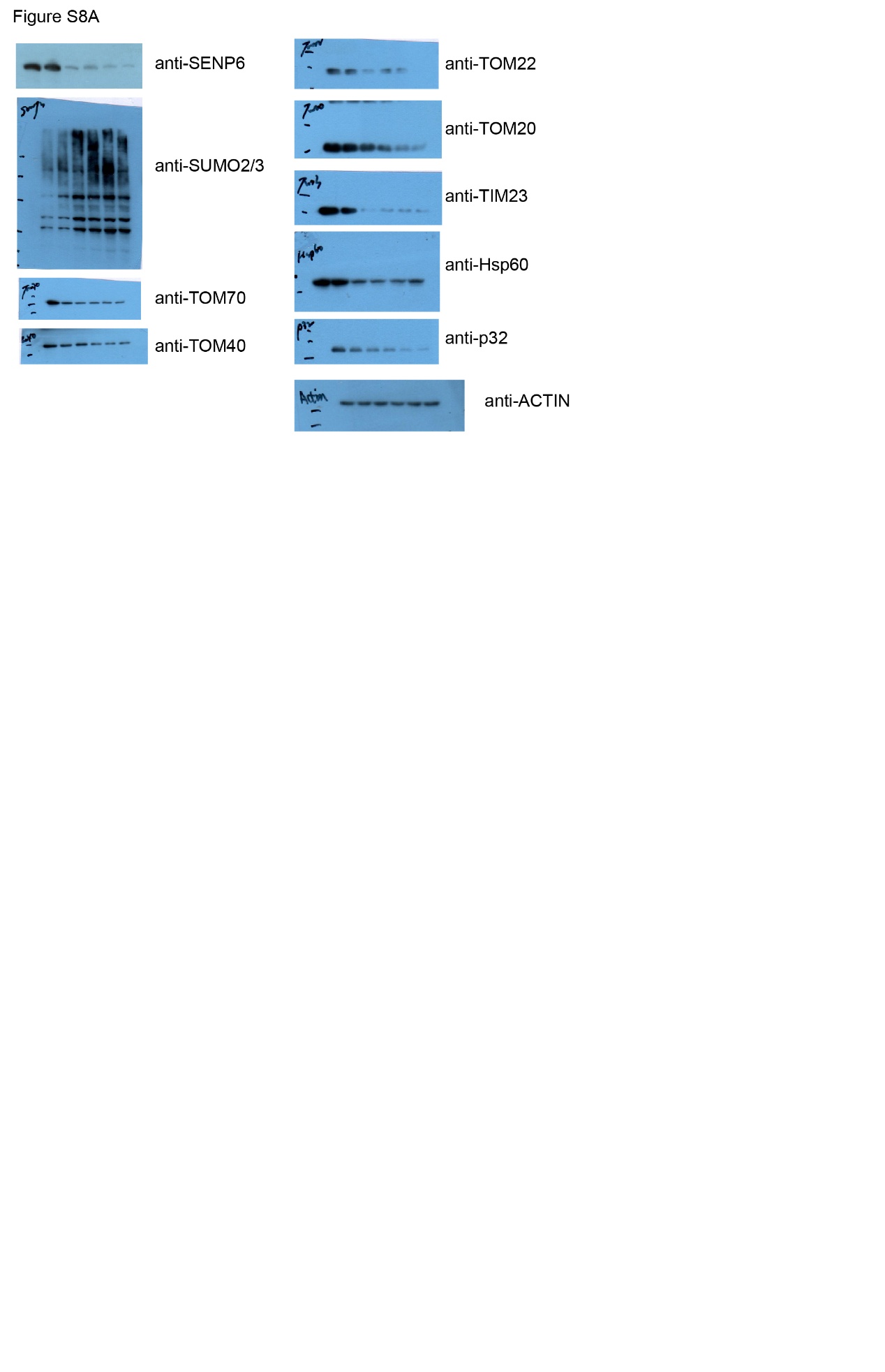
**

**Figure S9**

**
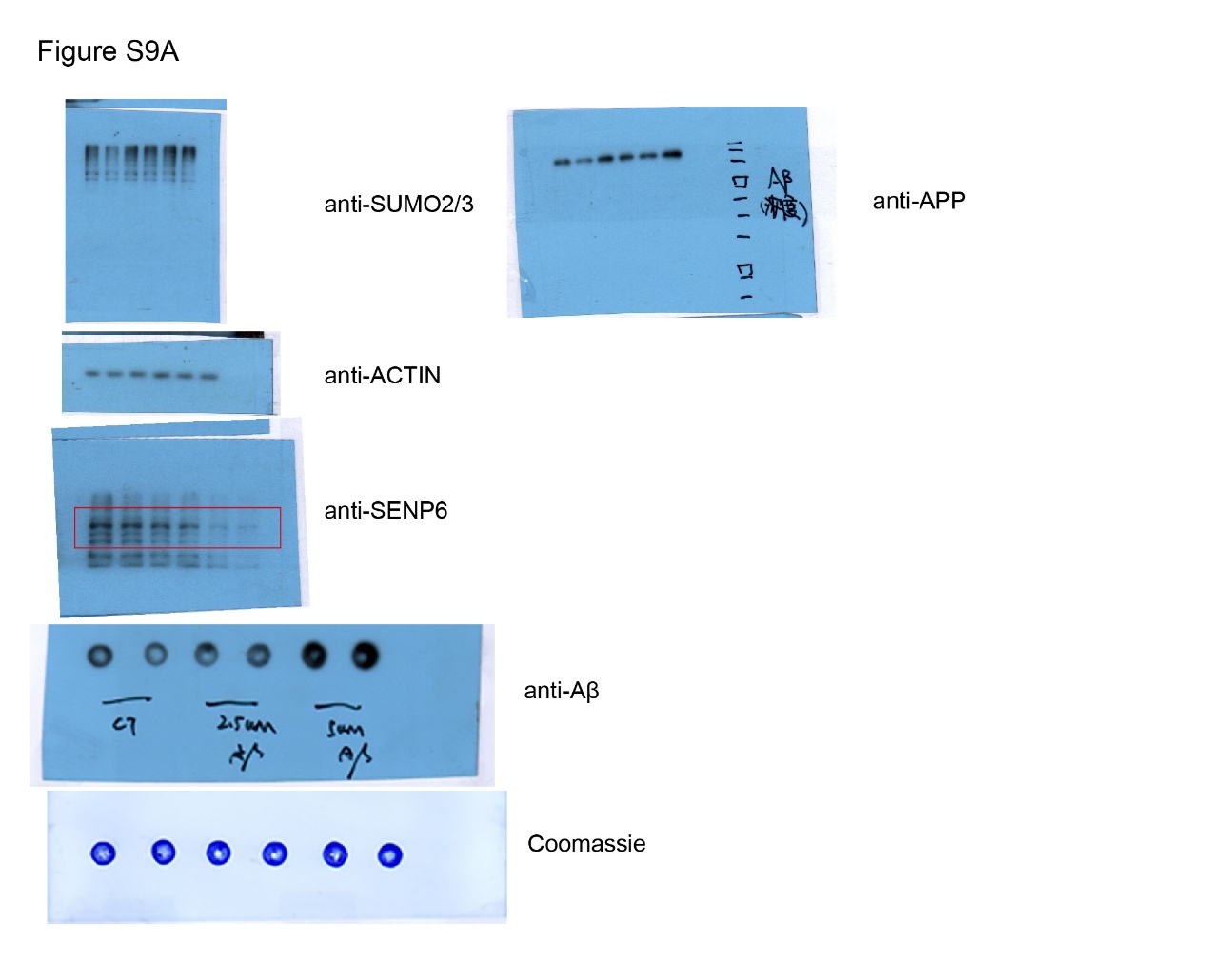
**

**Figure S10**

**
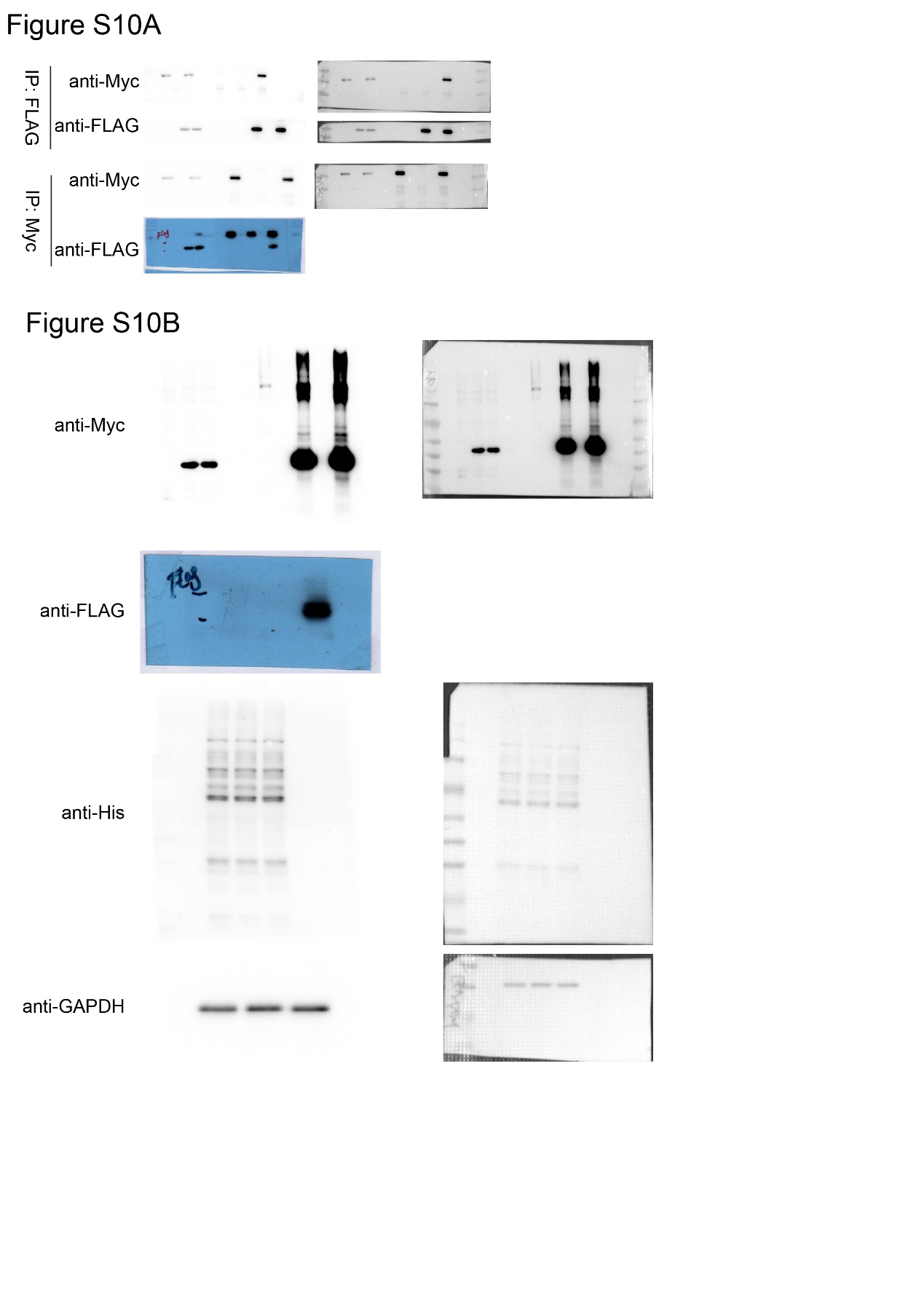
**
